# Comparison of total intravenous anesthesia and inhalational anesthesia in patients undergoing liver surgery: a systematic review and meta-analysis

**DOI:** 10.1016/j.bjane.2025.844604

**Published:** 2025-02-28

**Authors:** Gustavo R.M. Wegner, Bruno F.M. Wegner, Henrik G. Oliveira, Luis A. Costa, Luigi W. Spagnol, Valentine W. Spagnol, Jorge R.M. Carlotto, Eugénio Pagnussatt Neto

**Affiliations:** a Universidade Federal da Fronteira Sul (UFFS), Faculdade de Medicina, Passo Fundo, RS, Brazil; b Universidade Federal do Rio Grande do Sul (UFRGS), Faculdade de Medicina, Porto Alegre, RS, Brazil; c Universidade Federal da Fronteira Sul, Hospital de Clínicas, Departamento de Cirurgia, Passo Fundo, RS, Brazil; d Universidade Federal da Fronteira Sul (UFFS), Programa de Estágio Médico em Anestesiologia, Passo Fundo, RS, Brazil

**Keywords:** Anesthesia, Hepatectomy, Inhalational anesthesia, Liver transplant, Propofol

## Abstract

**Background:**

The impact of choosing between inhalational anesthetics and propofol for maintenance anesthesia in liver transplantation or liver resections remains uncertain.

**Methods:**

A systematic search was conducted on PubMed, Scopus, Embase, Web of Science, and the Cochrane Library on September 5, 2023, adhering to the Cochrane Handbook and PRISMA guidelines.

**Results:**

Fifteen randomized controlled trials and five observational studies, comprising 1,602 patients, were included. The statistical analysis was categorized into three groups: liver transplantation (four studies), living donor hepatectomy (four studies), and liver mass hepatectomy (twelve studies). The liver mass hepatectomy group was further subdivided based on the performance of the Pringle maneuver and the use of pharmacological preconditioning. Statistically significant results are described below. In liver transplant recipients, propofol anesthesia was associated with lower AST levels on the first postoperative day. Hepatic donors anesthetized with propofol had higher total infusion volumes and intraoperative urine output. Patients undergoing liver mass resection with the Pringle maneuver and propofol anesthesia had higher peak AST and ALT levels compared to those who received pharmacological preconditioning. Patients undergoing liver mass resection with the Pringle maneuver and propofol anesthesia had higher AST and ALT levels on both the first and third postoperative days, increased total infusion volumes, and shorter hospital stays, when compared to pharmacological conditioning.

**Conclusions:**

Our findings do not offer sufficient evidence to inform clinical practice. The choice between propofol-based and inhalational anesthesia should be tailored to the individual patient's condition and the nature of the procedure being performed.

**Registration:**

PROSPERO ID: CRD42023460715.

## Introduction

The efficacy and safety of anesthetic agents during surgical procedures, particularly in critical liver interventions, have been a subject of ongoing discussion in the medical community.^1^ The choice between propofol and inhaled anesthetics for maintaining anesthesia during hepatectomies is a critical decision, as it not only impacts intraoperative stability but also holds significant implications for postoperative liver function.^2^

Propofol exhibits anti-inflammatory properties, which contribute to the attenuation of the postoperative inflammatory response and the potential preservation of immediate liver function.^3^, ^4^, ^5^, ^6^, ^7^ Additionally, a recent article has linked the use of propofol for anesthetic maintenance during hepatectomies with a reduced incidence of postoperative liver dysfunction compared to inhaled anesthetics.^2^ On the other hand, another recent study investigating sevoflurane preconditioning in living liver donation found better initial graft function, highlighting the relevance of investigating the safety of inhalation anesthetics in living donors.^8^ Furthermore, in recent years, several studies have been published comparing the effects of inhalation anesthesia and propofol in liver mass resection surgeries, yet no clear advantage or disadvantage has been evidenced for either anesthetic agent.^2^^,^^9^^,^^10^

The published articles to date have not evidenced significant clinical differences between inhalation anesthetics and propofol in either liver transplant surgeries or hepatic resection surgeries. Therefore, our systematic review and meta-analysis aimed to compare inhalation anesthesia with propofol-based anesthesia on outcomes associated with postoperative liver enzymes tests and clinical outcomes such as hospital length of stay in patients undergoing liver transplant surgeries, whether as transplant recipients or liver donors, or liver mass resection surgeries.

## Methodology

### Design

The present systematic review investigated the effects of propofol versus inhalation anesthesia on liver function in liver transplant surgery and liver mass resection. The methodology was based on the Cochrane Handbook and the criteria suggested by PRISMA (Preferred Reporting Items for Systematic Reviews and Meta‐Analyses).^11^^,^^12^

#### Eligibility criteria

The inclusion criteria followed the Population-Intervention-Comparison-Outcome (PICO) principle, as follows: patients over 18-years old undergoing liver surgery (P), specifically comparing propofol (I) with an inhalational anesthetic (C), and evaluating liver function after the procedure (O). Both observational studies and randomized clinical trials were included.

#### Exclusion criteria

We excluded articles that specifically associated an intervention with any of the groups, such as propofol combined with dexmedetomidine versus inhalational anesthetics, or studies that combined inhalational anesthesia with propofol versus either propofol or inhalational anesthesia alone. We differentiated pharmacological preconditioning or conditioning from the use of inhalational anesthesia combined with propofol throughout the procedure. Articles that combined donor hepatectomy, hepatectomy for pathological indications, and liver transplantation into a single group were excluded. Articles published in languages other than English were also excluded.

No studies were excluded based on the type of pharmacological preconditioning, conditioning, or postconditioning. Articles presenting differences in the opioids used between the compared groups were not excluded.

### Search strategy

In this systematic review and meta-analysis, we searched PubMed, Scopus, Embase, Cochrane Library and Web of Science for reports published in English between date of database inception and September 5^th^, 2023.

The full search strategy consisted of: (“Hepatectomy” OR “Liver surgery”) AND (“Propofol” OR “intravenous anesthesia” OR “intravenous anaesthesia”) AND (“Sevoflurane” OR “Desflurane” OR “Isoflurane” OR “Volatile anesthetic” OR “Volatile anaesthetic” OR “inhalational anesthesia” OR “inhalational anaesthesia” OR “Inhaled anesthesia” OR “Inhaled anaesthesia” OR “Inhaled anesthetic” OR “Inhaled anaesthetic”).

Once the complete query terms were constructed, they were replicated in the Embase, Web of Science (all databases on the platform), Cochrane Library, Scopus, and PubMed electronic search engines.

The identified documents were exported to a reference manager (Mendeley 1.19.8®) to remove duplicates. The reference lists of all the included articles were also reviewed for potential citation eligibility.

### Selection of studies

Two reviewers (GRMW and BFMW) independently performed the two-step selection, screening studies based on titles and abstracts, followed by a full-text review of the articles selected in the first step. In cases of disagreement, a third reviewer (HGO) was consulted, and disagreements were resolved through consensus.

### Data extraction and synthesis

Data extraction was performed in duplicate using standardized data extraction tables in Google Sheets containing article identification, sample numbers and characteristics, and outcome measures. The data was collected in an inclusive and ostensive way and the relevant data was then synthesized by generating new tables for better comprehension. A meta-analysis was conducted when appropriate.

When data was described as recorded but inaccessible or difficult to extract, we contacted the corresponding author for data retrieval. For continuous data extracted from studies that only provided sample medians and ranges or first and third quartiles, calculations were used to estimate the sample mean and standard deviation.^13^^,^^14^ For data presented solely as images, WebPlotDigitizer 4.7 was employed to extract the relevant data.^15^ The images were uploaded into the software, and the axes were calibrated using known reference points. Data points were then manually digitized. The extracted data were cross-checked for accuracy and exported for analysis.

We contacted the corresponding authors of the studies by Koraki et al.,^9^ Laviolle et al.,^16^ Beck-Shimmer et al.,^17^ Ko et al.^18^ and Rodríguez et al.^19^ by email to clarify information related to the reporting of outcomes of interest and measures of variability, but we did not receive a response.

Statistical analyses were conducted using software *R*, employing random-effects with DerSimonian-Laird and inverse variance. The statistical analysis was performed considering significant heterogeneity for I^2^ values > 40% and statistical significance for a p-value < 0.05.

### Sensitivity analysis

We conducted a leave-one-out sensitivity analysis to ensure that the results of the meta-analysis were not overly dependent on any single study and to provide a clearer understanding of the variability and confidence of the findings. The result was considered consistent if there was no change in the direction of the effect, and heterogeneity did not shift from values exceeding 40% to below 40%, or from below 40% to exceeding 40%.

### Risk of bias assessment

For randomized clinical studies, the Cochrane Foundation Risk of Bias Assessment Tool was used, using the criteria of the RevMan software (5.4).^20^ The ROBINS-I (Risk of Bias In Non-randomized Studies ‒ of Interventions) tool was utilized to evaluate the risk of bias in non-randomized studies.^21^

## Results

**Figure 1:**
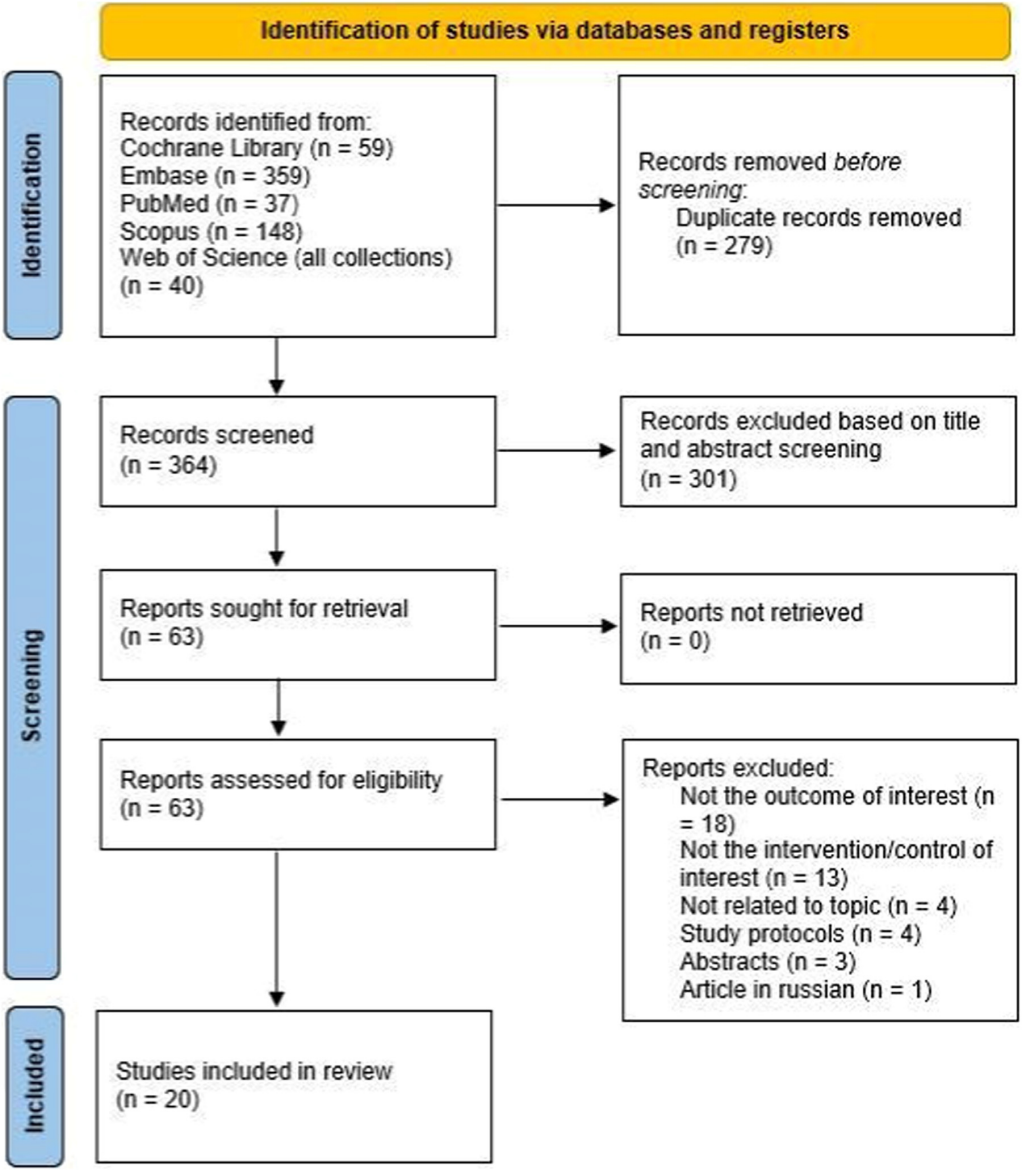
Study flow diagram.

Surveys were conducted on September 5^th^, 2023. A new search was conducted before the submission to the journal, with no new studies within our eligibility criteria identified. The selection process included 364 manuscripts, as presented by the PRISMA flowchart (Fig. 1). After all selection steps, 20 studies were included, 15 randomized controlled trials and 5 observational studies, with a total of 1,602 patients, of whom 808 received propofol, 524 sevoflurane, 166 desflurane and 104 isoflurane.^2^^,^^9^^,^^10^^,^^16^, ^17^, ^18^, ^19^^,^^22^, ^23^, ^24^, ^25^, ^26^, ^27^, ^28^, ^29^, ^30^, ^31^, ^32^, ^33^ Study characteristics are present in Table 1.

**Table 1:** Baseline characteristics of included studies.

Author	Year	Type	Pop	P (n) / Control (n)	Male %	Age (y)	ASA
Beck-Shimmer et al.	2008	RCT	LMR	P (34)/ S (30)	57.6/53.3	57.82 [12.82] / 54.2312.74]	1-3
Beck-Shimmer et al.	2015	RCT	LTR	P (48)/ S (50)	85/66	53 (37-61) / 58 (51-64)	3-4
Gajate et al.	2016	OBS	LTR	P (143) / S (58)	74.8/84.5	53.3 [8.4] / 54 [7.8]	–
Kamel et al.	2022	RCT	LMR	P (25)/ S (25)	‒	55 (51-59) / 56 (50-60)	2
Ko et al.	2008	RCT	LTD	P (35)/ D (35)	68.5/57.1	30.6 [10.9] / 28.8 [8.6]	1-2
Koraki et al.	2020	RCT	LMR	P (23)/ D (23)	52.1/60.8	61.5 [11.4] / 64.5 [10.6]	–
Laviolle et al.	2011	RCT	LMR	P (17)/ D (13)	82.3/46.1	60 [14] / 61 [13]	–
Lisnyy et al.	2023	OBS	LMR	P (32)/ S (41)	46.8/60.9	58 [7.4] / 58 [6.4]	2-3
Lu et al.	2014	OBS	LTR	P (66)/ D (45)	80.3/82.2	52.4 [7.9] / 53.5 [8.3]	–
Matsumi et al.	2023	RCT	LMR	P (28)/ S (28)	67.9/71.4	66.3 [12.3] / 64.7 [10.1]	2-3
Nguyen et al.	2019	OBS	LMR	P (26)/ S (67)	58/51	63 (53-68) / 61 (51-67)	–
Ozgul et al.	2013	RCT	LTD	P (40)/ I (40)	52.5/55	31 (19-48) / 33 (18-61)	1
Rabie et al.	2006	RCT	LTD	P (10)/ I (10)	80/90	24.6 [4.5] / 26.8 [5.3]	1
Rodríguez et al.	2015	RCT	LMR	P (36)/ S (34)	59/67	62[20] / 65 [12]	1-3
Slankamenac et al.	2012	OBS	LMR	P (86)/ S (141)	57.0/62.4	56.3 [12.7] / 59.2 [14.8]	1-4
Song et al.	2010	RCT	LMR	P (50)/ S (50)	72/80	51.4 [7.8] / 48.5 [8.9]	1-3
Ucar et al.	2015	RCT	LTD	P (29)/ I (24)	51.7/58.3	30.7 [7.8] / 35.4 [10.5]	1-2
Wu et al.	2019	RCT	LTR	P (25)/ D (25)	72/76	52.0 [9.1] / 53.2 [8.0]	–
Yang et al.	2010	RCT	LMR	P (30)/ I (30)	70/73.3	53.6 [9.5] / 52 [9.1]	2-3
Yassen et al.	2014	RCT	LMR	P (25)/ D (25)	96/72	55.2 [12.1] / 53.6 [10.4]	–

Pop, Population; LTR, Liver Transplant Recipient; P, Propofol; S, Sevoflurane; RCT, Randomized Controlled Trial; LMR, Liver Mass Resection; LTD, Liver Transplant Donor; D, Desflurane; I, Isoflurane. Mean [SD]; Median (IQR/Range).

A unified statistical analysis was not conducted due to substantial differences among the included studies. These differences stemmed from distinct patient populations, including liver transplant recipients, liver donors undergoing hepatectomy, and patients undergoing hepatectomy for hepatic masses. Additionally, techniques such as the Pringle maneuver and pharmacological preconditioning required stratification due to their significant impact on hepatic ischemia.^2^^,^^34^^,^^35^

Pharmacological preconditioning, conditioning, and postconditioning were categorized based on established academic definitions.^9^^,^^10^^,^^35^, ^36^, ^37^, ^38^, ^39^, ^40^, ^41^ Preconditioning refers to exposure to halogenated anesthetics before ischemia, conditioning involves continuous exposure throughout the anesthetic period, and postconditioning is defined as exposure to halogenated anesthetics exclusively after the ischemic period. Studies employing these distinct approaches were analyzed separately to preserve methodological rigor.

Variations in anesthetic regimens and adjunctive drugs among the studies were considered. However, these differences were not deemed substantial enough to invalidate or render the conducted analyses inappropriate.

Therefore, our statistical analysis was divided into three groups: liver transplantation (with four studies included), living donor hepatectomy (four studies), and liver mass hepatectomy (twelve studies).

### Liver transplant recipients

Four studies were included in the analysis of liver transplantation.^17^^,^^22^^,^^23^^,^^32^ We provide a detailed description of the included studies in the supplementary material. Supplementary Table 1 outlines data on anesthetic induction, maintenance, and pharmacological conditioning, while Supplementary Table 2 includes information on the indication for transplantation, patient age, MELD score, operative time, warm ischemia time, cold ischemia time, and donor type in liver transplantation.

All studies analyzed implemented pharmacological conditioning and postconditioning of the liver graft. The similarities among the studies were deemed sufficient to support statistical analysis.

All the outcomes with intersections across studies were evaluated. ALT levels on postoperative day 1 were the only hepatic enzyme parameter analyzed, and it was the sole outcome to show statistical significance, favoring the propofol group. However, as this analysis included only two studies, the possibility of a type 1 error must be considered. This limitation underscores the need for caution in interpreting the finding, which alone is insufficient to guide clinical practice regarding anesthetic agent selection.

The results are shown in Table 2 and the forest plots corresponding to each analysis are available in the supplementary material.

**Table 2:** Propofol versus inhalational anesthesia in liver transplant patients.

Propofol versus inhalational anesthesia in liver transplant patients						
Outcome	N (Studies)	Relative effect (95% CI)	p-value	I^2^ (%)	Leave-one-out^a^	Analysis plots
ALT 1^st^ postoperative day	161 (2)	MD=-131.09 U.L^−1^ (-212.41 to -49.78)	0.0016	0	‒	Supplementary Figure 1
Length of hospital stay (days)	259 (3)	MD=-1.84 days (-4.66 to 0.98)	0.2010	0	The results were consistent and not dependent on any single study.	Supplementary Figure 2 and 3
Length of ICU stay (hours)	259 (3)	MD=- 8.75 hours (-22.98 to 5.48)	0.2283	7	Heterogeneity was dependent on a single study.	Supplementary Figure 4 and 5
PRBC units	460 (4)	MD=1.32 units (- 0.40 to 3.04)	0.1311	82	Statistical significance was dependent on a single study.	Supplementary Figure 6 and 7
FFP units	259 (3)	MD=-1.61 units (-6.00 to 2.78)	0.4717	91	Heterogeneity and statistical significance were dependent on a single study.	Supplementary Figure 8 and 9
Platelets units	259 (3)	MD=0.17 units (-0.22 to 0.57)	0.3885	53	Heterogeneity and statistical significance were dependent on a single study.	Supplementary Figure 10 and 11
Total fluid infusion (L)	161 (2)	MD=0.29 L (-0.26 to 0.85)	0.3018	0	‒	Supplementary Figure 12
Estimated blood loss (L)	161 (2)	MD=0.50 L (-0.40 to 1.39)	0.2772	45	‒	Supplementary Figure 13
Urine output (L)	161 (2)	MD=0.13 L (-0.16 to 0.42)	0.3787	42	‒	Supplementary Figure 14
Early allograft dysfunction	299 (2)	RR=1.09 (0.52 to 2.28)	0.8283	65	‒	Supplementary Figure 15

MD, Mean Difference; PRBC, Packed Red Blood Cell; FFP: Fresh Frozen Plasma.

a The result was deemed consistent if no change in the direction of the effect occurred, and heterogeneity (I^2^) did not transition from values exceeding 40% to below 40% or from below 40% to exceeding 40%.

### Liver donor hepatectomy

Four studies were included in the analysis.^18^^,^^24^^,^^25^^,^^31^ We provide a detailed description of the included studies in the supplementary material. Supplementary Table 3 outlines data on anesthetic induction, maintenance, and pharmacological conditioning, while Supplementary Table 4 includes information on ASA, patient age, operative time, total liver volume, graft liver volume, remnant liver volume, and surgery. All studies analyzed implemented pharmacological conditioning.

We opted not to perform a statistical analysis across all studies comparing propofol and inhalational anesthesia in liver donors. We excluded Ko et al.'s^18^ study from statistical analysis as it was the only one that neither employed the Pringle maneuver nor blood flow occlusion, and we excluded Rabie et al.'s^25^ study due to the lack of information regarding whether the Pringle maneuver or blood flow occlusion was performed during hepatectomy.

All outcomes with intersections across studies were assessed. Only outcomes related to intraoperative fluid management demonstrated intersections. The results are summarized in Table 3, and the corresponding forest plots for each analysis are provided in the supplementary material.

**Table 3:** Propofol versus inhalational anesthesia in liver donor hepatectomy.

Propofol versus inhalational anesthesia in liver donor hepatectomy						
Outcome	n (Studies)	Relative effect (95% CI)	p-value	I^2^ (%)	Leave-one-out^a^	Analysis plots
Total fluid infusion (mL)	133 (2)	MD=673.73 mL (151.19 to 1196.26)	0.0115	0	‒	Supplementary Figure 16
Estimated blood loss (mL)	133 (2)	MD=-21.71 mL (-87.23 to 43.81)	0.5161	43	‒	Supplementary Figure 17
Urine output (mL)	133 (2)	MD=324.91 mL (60.13 to 589.49)	0.0162	0	‒	Supplementary Figure 18

MD, Mean Difference.

a The result was deemed consistent if no change in the direction of the effect occurred, and heterogeneity (I²) did not transition from values exceeding 40% to below 40% or from below 40% to exceeding 40%.

Total fluid infused and urine output were statistically higher in the propofol group. However, as this analysis included only two studies, the potential for a type I error must be considered.

### Liver mass resection

Twelve studies evaluated the difference between propofol and inhalational anesthetics in hepatectomy.^2^^,^^9^^,^^10^^,^^16^^,^^19^^,^^26^, ^27^, ^28^, ^29^, ^30^^,^^33^^,^^35^ Of these, ten studies performed the Pringle maneuver,^2^^,^^9^^,^^10^^,^^16^^,^^19^^,^^26^, ^27^, ^28^^,^^33^^,^^35^ one study neither performed blood flow occlusion nor the Pringle maneuver,^29^ and one study did not report whether the Pringle maneuver or blood flow occlusion was conducted.^30^

Four studies exclusively employed pharmacological preconditioning,^9^^,^^19^^,^^33^^,^^35^ another employed both pharmacological conditioning and preconditioning,^10^ while seven studies reported the use of pharmacological conditioning.^2^^,^^16^^,^^26^, ^27^, ^28^, ^29^, ^30^

We consider that meta-analyses aggregating studies employing the Pringle maneuver with those that did not are methodologically inappropriate. Similarly, aggregating studies that utilized pharmacological conditioning with those employing pharmacological preconditioning is also unsuitable.

Therefore, we grouped the studies based on the following criteria: 1) Similarity in surgical approach (Pringle maneuver/blood flow occlusion), 2) Use of pharmacological conditioning or pharmacological preconditioning, 3) No significant discrepancies in anesthetic maintenance regimens.

We provide a detailed description of the studies evaluating the difference between propofol and inhalational anesthetics in hepatectomy in the supplementary material. Supplementary Table 5 presents data on anesthetic induction, maintenance, and pharmacological conditioning. Supplementary Table 6 details patient characteristics, including ASA classification, age, operative time, ischemia duration, presence of cirrhosis, baseline levels of AST, ALT, and bilirubin, surgical indication, and the extent of hepatectomy.

### Hepatic mass resection without the Pringle maneuver and with pharmacological conditioning

Two studies performed pharmacological conditioning and did not describe the Pringle maneuver or blood flow occlusion.^29^^,^^30^ While Yassen et al.^29^ did not use either the Pringle maneuver or blood flow occlusion, there is uncertainty regarding the use of these techniques in the study by Kamel et al.^30^ Therefore, we chose not to perform a pooled statistical analysis combining the data from Kamel et al. and Yassen et al.

### Hepatic mass resection with the Pringle maneuver and pharmacological preconditioning

Five studies performed pharmacological preconditioning and described the Pringle maneuver.^9^^,^^10^^,^^19^^,^^33^^,^^35^ However, the study by Lisnyy et al.^33^ reports the use of the Pringle maneuver in only a subset of the patients studied and does not provide stratified data distinguishing between patients who underwent the Pringle maneuver and those who did not. As a result, it was not included in the statistical analysis. The similarities among the other studies were deemed sufficient to support statistical analysis.

All outcomes overlapping in two or more studies were analyzed. Specifically, we examined peak AST, peak ALT, peak bilirubin, estimated blood loss during the procedure, and hospital length of stay. The results of the analyses are described in Table 4.

**Table 4:** Propofol versus inhalational anesthesia in liver mass resection with pharmacological preconditioning.

Propofol versus inhalational anesthesia in liver mass resection with pharmacological preconditioning						
Outcome	n (Studies)	Relative effect (95% CI)	p-value	I^2^ (%)	Leave-one-out^a^	Analysis plots
Peak AST	233 (4)	MD=141.77 U.L^−1^ (42.53 to 241)	0.0051	56	Statistical significance was dependent on a single study.	Supplementary Figure 19 and 20
Peak ALT	233 (4)	MD=107.07 U.L^−1^ (30.85 to 183.28)	0.0059	49	Heterogeneity was dependent on a single study. Statistical significance was dependent on single studies.	Supplementary Figure 21 and 22
Peak Bilirubin	180 (3)	MD=-0.18 mg.dL^−1^ (-0.52 to 0.15)	0.2859	0	The results were consistent and not dependent on any single study.	Supplementary Figure 23 and 24
Length of hospital stay (days)	233 (4)	MD=-1.23 days (-3.65 to 1.19)	0.3181	90	The results were consistent and not dependent on any single study.	Supplementary Figure 25 and 26
Estimated blood loss (mL)	169 (3)	MD=24.70 mL (-91.86 to 141.25)	0.6779	75	Heterogeneity was dependent on single studies. Statistical significance was dependent on a single study.	Supplementary Figure 27 and 28

MD, Mean Difference.

a The result was deemed consistent if no change in the direction of the effect occurred, and heterogeneity (I^2^) did not transition from values exceeding 40% to below 40% or from below 40% to exceeding 40%.

Although the study by Koraki et al.^9^ overlapped with Nguyen et al.'s^10^ study in reporting AST and ALT levels during the early postoperative days, we chose not to analyze them together due to inconsistencies in data presentation by Koraki et al.^9^ Similarly, it was not possible to extract data regarding AST and ALT levels during the early postoperative days from Rodríguez et al.'s article.^19^ We contacted the corresponding author of Koraki et al.'s and Rodríguez et al. study but did not receive a response.

Propofol was associated with statistically higher peaks in AST and ALT. No statistically significant difference was observed in peak bilirubin levels, hospital length of stay and estimated blood loss. Despite the higher aminotransferase peaks in the postoperative period in the propofol group, the clinical implications of these findings remain uncertain, suggesting that further studies are necessary to guide clinical practice. Furthermore, the potential for a type I error must be considered in all statistically significant outcomes.

### Hepatic mass resection with the Pringle maneuver and pharmacological conditioning

Six studies implemented pharmacological conditioning and reported the Pringle maneuver.^2^^,^^10^^,^^16^^,^^26^, ^27^, ^28^ The similarities among these studies were considered sufficient to justify statistical analysis; nevertheless, we present a stratified analysis based on the presence of cirrhosis.

All outcomes overlapping in two or more studies were analyzed. Specifically, we examined peak AST, peak ALT, peak bilirubin, AST and ALT levels on the first and third postoperative days, total fluids infused during the intraoperative period, estimated blood loss, and hospital length of stay. The results of the analyses are described in Table 5.

**Table 5:** Propofol versus inhalational anesthesia in liver mass resection.

Propofol versus inhalational anesthesia in liver mass resection						
Outcome	n (Studies)	Relative effect (95% CI)	p-value	I^2^ (%)	Leave-one-out^a^	Analysis plots
Peak AST	519 (5)	MD=39.70 U.L^−1^ (-75.12 to 154.52)	0.4980	74	Heterogeneity and statistical significance were dependent on a single study.	Supplementary Figures 29 and 30
Peak ALT	519 (5)	MD=3.20 U.L^−1^ (-104.46 to 110.85)	0.9536	71	Heterogeneity and statistical significance were dependent on a single study.	Supplementary Figures 31 and 32
Peak Bilirubin	443 (4)	MD=0.11 mg.dL^−1^ (-0.20 to 0.42)	0.4844	51	Heterogeneity was dependent on a single study.	Supplementary Figures 33 and 34
AST 1^st^ postoperative day	253 (3)	MD=94.08 U.L^−1^ (56.10 to 132.06)	< 0.0001	29	Heterogeneity was dependent on single studies.	Supplementary Figures 35 and 36
AST 3^rd^ postoperative day	253 (3)	MD=18.00 U.L^−1^ (3.91 to 32.08)	0.0123	48	Statistical significance was dependent on single studies.	Supplementary Figures 37 and 38
ALT 1^st^ postoperative day	253 (3)	MD=71.70 U.L^−1^ (40.23 to 101.97)	< 0.0001	8	The results were consistent and not dependent on any single study.	Supplementary Figures 39 and 40
ALT 3^rd^ postoperative day	253 (3)	MD=18.11 U.L^−1^ (2.23 to 33.99)	0.0254	0	Statistical significance was dependent on single studies.	Supplementary Figures 41 and 42
Length of hospital stay (days)	480 (4)	MD=-1.17 days (-2.32 to -0.02)	0.0455	65	Heterogeneity was dependent on a single study. Statistical significance was dependent on single studies.	Supplementary Figures 43 and 44
Total fluid infusion (mL)	153 (2)	MD=286.22 mL (171.89 to 400.55)	< 0.0001	0	‒	Supplementary Figure 45
Estimated blood loss (mL)	566 (6)	MD=12.64 mL (-59.29 to 84.57)	0.7305	59	Heterogeneity was dependent on a single study.	Supplementary Figures 46 and 47

MD, Mean Difference.

a The result was deemed consistent if no change in the direction of the effect occurred, and heterogeneity (I^2^) did not transition from values exceeding 40% to below 40% or from below 40% to exceeding 40%.

While ALT and AST levels on the first and third postoperative days, as well as the total fluids infused during the procedure, were statistically higher in the propofol group, hospital length of stay was statistically shorter in the propofol group. No statistically significant differences were found in the other outcomes analyzed. Despite the higher aminotransferase levels in the postoperative period in the propofol group, the clinical implications of these findings remain uncertain, indicating that further studies are needed to guide clinical practice. Furthermore, the potential for a type I error must be considered in all statistically significant results.

### Risk of bias assessment

The Risk of Bias 2 (RoB 2) tool was used for quality assessment in randomized clinical trials and the ROBINS-I for non-randomized studies.^20^^,^^21^ The assessment of bias can be found in the supplementary material, specifically in Supplementary Figure 48.

## Discussion

This study provides a systematic review and meta-analysis comparing propofol with inhalation anesthesia in liver surgery. 1) Liver transplant recipients anesthetized with propofol had statistically lower AST levels on the first postoperative day. 2) Hepatic donors anesthetized with propofol had statistically higher total infusion volumes and intraoperative urine output. 3) Patients undergoing liver mass resection with the Pringle maneuver and propofol anesthesia had statistically higher AST and ALT peaks compared to those receiving pharmacological preconditioning. 4) Patients undergoing liver mass resection with the Pringle maneuver and propofol anesthesia had statistically higher AST and ALT levels on the first and third postoperative days, greater total infusion volume, and shorter hospital stays compared to those undergoing pharmacological conditioning. There were no statistically significant differences observed in the other outcomes analyzed.

The observed statistically significant findings are insufficient to guide clinical practice. The absence of statistically significant changes in other liver function and enzymatic tests, which would substantiate and lend consistency to these results, underscores the need for further research to optimize perioperative care in patients undergoing liver surgery.

### Liver transplant patients

#### Liver transplant recipients

Our analysis identified only a statistically significant difference favoring propofol in ALT levels on the first postoperative day. Regarding other outcomes not included in our statistical analysis, the study by Lu et al.^23^ demonstrated that propofol provided advantages over desflurane during the hepatic reperfusion period, including a reduced need for rescue vasoactive medications and a lower total dose of these drugs. Similarly, Wu et al.^22^ observed a more favorable inflammatory response with propofol compared to desflurane. Despite these findings, neither study reported the occurrence of early allograft dysfunction, leaving this outcome unexplored in their analyses.

The study conducted by Beck-Schimmer et al.^17^ suggested that post-conditioning with sevoflurane might offer advantages over propofol in cadaveric liver transplant recipients by reducing early allograft dysfunction and mitigating severe complications. However, in their study, the results for these outcomes were not statistically significant, and in the comparative statistical analysis with the study by Gajate et al., no statistically significant findings were observed either.

In a recent study, Dieu et al.^8^ demonstrated the advantages of pre-conditioning with sevoflurane during donor surgery in living donor liver transplants, showing a significant reduction in early allograft dysfunction in pediatric liver transplant recipients. Similarly, Minou et al.^42^ reported a significant advantage of pre-conditioning with sevoflurane in deceased donors regarding the incidence of early allograft dysfunction in liver transplant recipients, though no differences in hepatic inflammation tests were observed. The study by Minou et al.^42^ was not included in our analysis, despite comparing the use of inhalational anesthetics and propofol, as its evaluation of hepatic function in transplant recipients based on the anesthetic used for the donor was not within the scope of our review.

The definition of early allograft dysfunction varies among studies. Gajate et al.'s^32^ study differs from those of Dieu et al.,^8^ Minou et al.,^42^ and Beck-Schimmer et al.^17^ in their definition of early allograft dysfunction, using only transaminases as outcome markers. While the analysis of early allograft dysfunction based on the type of hypnotic agent used by the donor is essential, the studies included in our analysis, Beck-Schimmer et al.,^17^ Lu et al.,^23^ and Wu et al.,^22^ did not describe or investigate the conditions under which donor liver surgeries were performed, thereby preventing any statistical evaluation of this factor.

Beck-Schimmer et al.^17^ proposed that prolonged cold ischemia time might have obscured potential advantages of using halogenated anesthetics. However, Wu et al.'s^22^ study, which reported shorter cold ischemia times, also failed to identify any benefits, suggesting that the hypothesis of cold ischemia time mitigating or masking the effects of halogenated anesthetics is not supported by the available evidence. If cold ischemia times such as those reported by Wu et al.^22^ indeed diminish the hypothetical effects of halogenated anesthetics, the rationale for their use to mitigate ischemia-reperfusion injury in liver transplantation becomes questionable. Furthermore, the comparable outcomes with propofol suggest that the effect size of halogenated anesthetics might be similar to that of propofol.

#### Living donor hepatectomy

Our analysis of living transplant donors included studies by Ko et al.,^18^ who used desflurane for anesthesia maintenance, and Ozgul et al.,^24^ Rabie et al.,^25^ and Ucar et al.,^31^ who used isoflurane for anesthesia maintenance. None of these studies evaluated early allograft dysfunction in their respective recipients. Only Ozgul et al.^24^ and Ucar et al.^31^ were statistically analyzed. The limitation of our analysis to intraoperative fluid management precludes its findings from guiding perioperative management in liver donor hepatectomy.

Future studies should stratify using the Pringle maneuver and evaluate early allograft dysfunction in recipients to determine whether donor anesthetics influence outcomes. When inhalational anesthetics are used during donor hepatectomy, pharmacological preconditioning occurs, whereas their use exclusively in recipients constitutes pharmacological postconditioning of the graft. The hypothesis that inhalational anesthetics may improve hepatic outcomes requires investigation in both contexts. Our analysis does not provide sufficient evidence to confirm or refute either hypothesis.

#### Liver mass resection

It was not possible to evaluate the differences between the use of propofol and inhalational anesthetics in liver resections performed without the Pringle maneuver. Yassen et al.^29^ did not identify significant differences between these anesthetic approaches. A key point highlighted in their study is that avoiding the Pringle maneuver eliminates ischemia-reperfusion injury, an operative context in which the effects of propofol and inhalational anesthetics remain uncertain.

We recognize that the discussion regarding the comparison between propofol and pharmacological preconditioning, as well as the comparison between propofol and ischemic preconditioning, must be addressed separately.

### Propofol versus pharmacological preconditioning

Propofol demonstrated higher peaks of AST and ALT. The clinical implications of our findings are limited due to the small number of included studies and patients, as well as the restricted range of outcomes analyzed. Currently, there is no evidence that the observed differences translate into significant clinical effects. Nonetheless, critical points for discussion emerge from the studies conducted thus far.

Regarding pharmacological preconditioning, several considerations must be addressed. There remains uncertainty about potential modifying effects associated with the strategy when performed in conjunction with clamping or the Pringle maneuver.^9^^,^^10^^,^^19^^,^^35^ Three modes of clamping the portal triad are described in the literature: continuous clamping, intermittent clamping (characterized by cycles of 15 minutes of clamping followed by 5 minutes of reperfusion), and ischemic preconditioning, which involves 5-15 minute cycles of ischemia and reperfusion conducted prior to the main ischemic period.^43^, ^44^, ^45^ In our study, all three types of clamping were classified as the Pringle maneuver, and clamping methods did not differ among the analyzed groups.

There is evidence that intermittent clamping and ischemic preconditioning can reduce ischemia-reperfusion injury.^46^, ^47^, ^48^, ^49^ Regarding ischemic preconditioning, a systematic review and meta-analysis identified lower blood loss, reduced transfusion requirements, and decreased postoperative ascites in the ischemic preconditioning group compared to continuous or intermittent clamping.^43^ However, there was no evidence that ischemic preconditioning attenuated ischemia-reperfusion injury compared to the other clamping methods.^43^

This context frames the contrast between the studies by Rodríguez et al.^19^ and Nguyen et al.,^10^ which did not identify statistically significant advantages for pharmacological preconditioning under intermittent clamping concerning aminotransferase levels, and the study by Koraki et al.,^9^ which demonstrated advantages for pharmacological preconditioning under intermittent clamping concerning aminotransferase levels. Furthermore, the study by Beck-Schimmer et al.^35^ showed advantages for pharmacological preconditioning in the setting of continuous clamping, while the study by Lisnyy et al.^33^ identified benefits of pharmacological preconditioning in a heterogeneous cohort, in which some patients received intermittent clamping, whereas others were not subjected to clamping.

Currently, no studies have investigated the dose-dependent effects or the impact of exposure duration to halogenated anesthetics in clinical settings, although evidence from animal and in vitro research suggests that these factors may be significant.^10^^,^^50^, ^51^, ^52^ While Beck-Schimmer et al.,^35^ Lisnyy et al.,^33^ Nguyen et al.,^10^ and Koraki et al.^9^ performed pharmacological preconditioning for 30 minutes, Rodríguez et al.^19^ conducted preconditioning for 20 minutes. Thus, there is uncertainty not only regarding the optimal duration of preconditioning but also concerning the doses used.

Additionally, there is ongoing discussion regarding whether halogenated anesthetics mimic the effects of ischemic preconditioning.^10^^,^^53^^,^^54^ Nevertheless, the study by Rodríguez et al.^19^ demonstrated superior outcomes with pharmacological preconditioning compared to ischemic preconditioning. Their findings showed that the group subjected to ischemic preconditioning had higher aminotransferase levels compared to those who underwent pharmacological preconditioning or received propofol alone.

### Propofol versus pharmacological conditioning

The conflicting findings of propofol being associated with shorter hospital stays but higher postoperative aminotransferase levels underscore the need for further studies to clarify the differences between propofol and inhalational anesthetics in hepatic resection surgeries.

A more detailed analysis of patients at higher risk for postoperative liver failure, based on factors described by Orozco et al.,^34^ such as extent of hepatectomy, hepatectomy due to metastasis, and transfusion within 72 hours, as well as factors identified as effect modifiers in the Beck-Shimmer et al.^35^ study, including fibrosis and hepatic steatosis, would have been valuable. This would help assess not only the impact of these factors on the effects of propofol and inhalational anesthesia but also how these interventions influence higher-risk patients. However, due to limited data availability, we were not able to incorporate these variables into our analysis.

In addition to tumor metastasis being an isolated risk factor for liver failure in the postoperative period in non-cirrhotic patients, there are additional reasons for stratifying the analysis based on the surgical indication for hepatectomy. A retrospective study involving 670 patients conducted between 2005 and 2014 revealed a significant increase in mortality among patients undergoing hepatectomy for hepatocellular carcinoma exposed to inhalational anesthesia with desflurane compared to propofol.^55^ Therefore, the use of inhalational anesthetics in hepatectomies, especially for malignant neoplasm resections, should be approached with caution.

Our study primarily has implications for future research. The high heterogeneity observed in clinical studies comparing anesthetic regimens in hepatic surgeries underscores the complexity of factors that may interfere with the interventions analyzed. Variables such as pre-existing liver dysfunction, the presence of malignancy or metastasis, and the type of Pringle maneuver employed must be carefully considered, as they ‘could influence perioperative outcomes. Future investigations of hepatectomies performed for pathological causes should stratify findings by specific etiological factors and the extent of hepatectomy, given their potential impact on the outcomes analyzed. Moreover, studies assessing pharmacological preconditioning should rigorously account for the type of Pringle maneuver applied, as this variable may modify the observed effects. Addressing these considerations is essential for improving perioperative care for patients undergoing hepatic surgeries.

## Limitations

Although our meta-analysis assembled several robust experimental models regarding the influence of the choice of anesthetic regimen on the postoperative hepatic function of patients undergoing hepatectomies, our analysis is subject to some limitations. Firstly, there was relative methodological heterogeneity among the included manuscripts, with disparities in terms of the clinical profiles of patients, the type of procedure, the anesthetic dose used, and the adjuvant medications added in multimodal anesthesia. Additionally, several factors needed to be stratified to make the analysis feasible, thereby limiting the power of our analysis to identify differences between interventions. Nevertheless, the statistically significant differences found are not sufficient to guide clinical practice.

## Conclusion

Our findings do not show significant clinical differences between the use of propofol-based anesthesia and inhaled anesthetics. The choice between the use of inhalational anesthetics and propofol-based anesthesia should be individualized for each patient and procedure performed.

## Authors’ contributions

The authors confirm contribution to the paper as follows: study conception and design: Gustavo R.M. Wegner; data collection: Gustavo R.M. Wegner, Henrik G. Oliveira; analysis and interpretation of results: Gustavo R.M. Wegner, Bruno F.M. Wegner, Luis A. Costa, Luigi W. Spagnol, Valentine W. Spagnol; draft manuscript preparation: Gustavo R.M. Wegner, Bruno F.M. Wegner, Henrik G. Oliveira, Luis A. Costa, Luigi W. Spagnol, Valentine W. Spagnol. Revision and guidance: Jorge R.M. Carlotto, Eugénio Pagnussatt Neto. All authors reviewed the results and approved the final version of the manuscript.

## Declaration of competing interest

The authors declare no conflicts of interest.

## Appendix Supplementary materials

Image, application 1

## Footnotes

Supplementary material associated with this article can be found in the online version at doi:10.1016/j.bjane.2025.844604.
