# Supplementary material for: Comparison of total intravenous anesthesia and inhalational anesthesia in patients undergoing liver surgery: a systematic review and meta-analysis
Source: Braz J Anesthesiol. 2025 Feb 28;75(3):844604. doi: 10.1016/j.bjane.2025.844604 (PMC11960546; doi:10.1016/j.bjane.2025.844604)

**BJAN-D-24-00342**

**Supplementary material**

1. **Supplementary tables**
   1. Supplementary table 1: Liver transplantation supplementary table
   2. Supplementary table 2: Baseline characteristics of patients undergoing liver transplant.
   3. Supplementary table 3: Liver mass resection supplementary table
   4. Supplementary table 4: Baseline characteristics of patients undergoing liver donor hepatectomy
   5. Supplementary table 5: Liver donor hepatectomy supplementary table
   6. Supplementary table 6: Baseline characteristics of patients undergoing liver mass resection
2. **Supplementary figures**
   1. Propofol versus inhalational anesthesia in liver transplant patients
      1. Supplementary figure 1: ALT 1st postoperative day
      2. Supplementary figure 2: Length of hospital stay (days)
      3. Supplementary figure 3: Length of hospital stay (days) - Leave-one-out
      4. Supplementary figure 4: Length of ICU stay (hours)
      5. Supplementary figure 5: Length of ICU stay (hours) - Leave-one-out
      6. Supplementary figure 6: Packed red blood cell units
      7. Supplementary figure 7: Packed red blood cell units - Leave-one-out
      8. Supplementary figure 8: Fresh Frozen Plasma units
      9. Supplementary figure 9: Fresh Frozen Plasma units - Leave-one-out
      10. Supplementary figure 10: Platelets units
      11. Supplementary figure 11: Platelets units - Leave-one-out
      12. Supplementary figure 12: Total fluid infusion (L)
      13. Supplementary figure 13: Estimated blood loss (L)
      14. Supplementary figure 14: Urine output (L)
      15. Supplementary figure 15: Early allograft dysfunction
   2. Propofol versus inhalational anesthesia in liver donor hepatectomy
      1. Supplementary figure 16: Total fluid infusion (ml)
      2. Supplementary figure 17: Estimated blood loss (ml)
      3. Supplementary figure 18: Urine output (ml)
   3. Propofol versus inhalational anesthesia in liver mass resection with pharmacological preconditioning
      1. Supplementary figure 19: Peak AST
      2. Supplementary figure 20: Peak AST - Leave-one-out
      3. Supplementary figure 21: Peak ALT
      4. Supplementary figure 22: Peak ALT - Leave-one-out
      5. Supplementary figure 23: Peak Bilirubin
      6. Supplementary figure 24: Peak Bilirubin - Leave-one-out
      7. Supplementary figure 25: Length of hospital stay (days)
      8. Supplementary figure 26: Length of hospital stay (days) - Leave-one-out
      9. Supplementary figure 27: Estimated blood loss (ml)
      10. Supplementary figure 28:Estimated blood loss (ml) - Leave-one-out
   4. Propofol versus inhalational anesthesia in liver mass resection
      1. Supplementary figure 29: Peak AST
      2. Supplementary figure 30: Peak AST - Leave-one-out
      3. Supplementary figure 31: Peak ALT
      4. Supplementary figure 32: Peak ALT - Leave-one-out
      5. Supplementary figure 33: Peak Bilirubin
      6. Supplementary figure 34: Peak Bilirubin - Leave-one-out
      7. Supplementary figure 35: AST 1st postoperative day
      8. Supplementary figure 36: AST 1st postoperative day - Leave-one-out
      9. Supplementary figure 37: AST 3rd postoperative day
      10. Supplementary figure 38: AST 3rd postoperative day - Leave-one-out
      11. Supplementary figure 39: ALT 1st postoperative day
      12. Supplementary figure 40: ALT 1st postoperative day - Leave-one-out
      13. Supplementary figure 41: ALT 3rd postoperative day
      14. Supplementary figure 42: ALT 3rd postoperative day - Leave-one-out
      15. Supplementary figure 43: Length of hospital stay (days)
      16. Supplementary figure 44: Length of hospital stay (days) - Leave-one-out
      17. Supplementary figure 45: Total fluid infusion (ml)
      18. Supplementary figure 46: Estimated blood loss (ml)
      19. Supplementary figure 47: Estimated blood loss (ml) - Leave-one-out
   5. Risk of bias assessment
      1. Supplementary figure 48: Risk of bias assessment

**1.1 Supplementary table 1: Liver transplantation supplementary table**

| **Author** | **Anesthesia  induction** | **Anesthesia maintenance** | | **Pharmacological conditioning* / Pringle maneuver**** |
| --- | --- | --- | --- | --- |
|  |  | **Propofol anesthesia group** | **Inhalational anesthesia group** |  |
| Beck-Shimmer et al. (2015)  RCT | Propofol TCI 3–6 mcg/ml (or 5–7 mcg/mL for rapid sequence induction).  Fentanyl: 3 mcg/kg IV (or initiation of remifentanil infusion).  Atracurium: 0.5 mg/kg IV.  Rocuronium: 0.9 mg/kg IV (for rapid sequence induction). | Propofol TCI 3-6 mcg/ml  BIS 40-60.  Fentanyl: 1–2 mcg/kg IV bolus (as needed).  Remifentanil: Continuous infusion up to 20 mcg/kg/h (as needed).  Atracurium: 5–10 mg IV (when Train-of-Four response ≥ 2). | Sevoflurane 0.6-1.5 MAC  BIS 40-60.  Fentanyl: 1–2 mcg/kg IV bolus (as needed).  Remifentanil: Continuous infusion up to 20 mcg/kg/h (as needed).  Atracurium: 5–10 mg IV (when Train-of-Four response ≥ 2). | * The recipient experienced pharmacological conditioning with sevoflurane through the entire procedure. The transplanted liver itself received a pharmacological postconditioning with sevoflurane.  -*** |
| Gajate et al. OBS | General anesthesia was  initiated using a rapid sequence induction, with fentanyl, suxamethonium, and propofol | Propofol  Remifentanil infusion to obtain optimal analgesia levels and nondepolarizing neuromuscular blocker agents were administered according to clinical needs. | Sevoflurane Remifentanil infusion to obtain optimal analgesia levels and nondepolarizing neuromuscular blocker agents were administered according to clinical needs. | * The recipient experienced pharmacological conditioning with sevoflurane through the entire procedure. The transplanted liver itself received a pharmacological postconditioning with sevoflurane.  -*** |
| Lu et al.  OBS | Propofol group:  Propofol TCI 3–5 mcg/ml.  Fentanyl: 1-2 mcg/kg IV.  Lidocaine: 1 mg/kg IV.  Rocuronium: 0.6 mg/kg IV. | Propofol TCI 3-5 mcg/ml  AAI 15-25.  Ce of propofol was reduced or increased by 2% and 0.5 mg/  mL, respectively, in order to keep the AAI within 15-25. | Desflurane in 100% oxygen with wash-in at 2 L/minute for 15 minutes, followed by a flow rate of 0.3 L/minute thereafter.  AAI 15-25.  Desflurane was reduced or increased by 2%, respectively, in order to keep the AAI within 15-25. | * The recipient experienced pharmacological conditioning with desflurane through the entire procedure. The transplanted liver itself received a pharmacological postconditioning with desflurane.  -*** |
|  | Inhalational anesthesia group:  Propofol 2 mg/kg IV.  Fentanyl: 1-2 mcg/kg IV.  Lidocaine: 1 mg/kg IV.  Rocuronium: 0.6 mg/kg IV. |  |  |  |
| Wu et al.  RCT | Propofol 1-2 mg/kg IV.  Fentanyl 1-2 mcg/kg IV.  Lidocaine 1 mg/kg IV.  Rocuronium 0.6 mg/kg IV. | “Propofol TCI was reduced or increased by 0.5 mcg/ml, respectively, in order to keep the BIS value between 40 and 60.” | “Desflurane was reduced or increased by 2%, respectively, in order to keep the BIS value between 40 and 60.” | * The recipient experienced pharmacological conditioning with desflurane through the entire procedure. The transplanted liver itself received a pharmacological postconditioning with desflurane.  -*** |

* Definition for pharmacological conditioning used: The cytoprotective effect of volatile anesthetics may either be initiated before the onset of ischemic injury (preconditioning), immediately on reperfusion (postconditioning), or for the entire surgical procedure (conditioning).

** The Pringle maneuver was included in our analyses as part of subgroup analyses to evaluate whether the use of inhalational anesthetics or propofol might exert a more pronounced effect in the context of ischemia-reperfusion. However, for studies that employed pharmacological preconditioning, we opted for stratified analyses, as the use of a specific anesthetic exclusively during the ischemic period could result in a modifying effect. Therefore, we did not jointly analyze studies that performed only the Pringle maneuver with those that combined the Pringle maneuver and pharmacological preconditioning. It is worth noting that in cases where pharmacological preconditioning was not specifically performed, continuous exposure to a single pharmacological agent throughout the surgical procedure after induction was considered pharmacological conditioning.

*** There is no description of whether the Pringle maneuver, any form of ischemic preconditioning, or inflow occlusion was performed.

**1.2 Supplementary table 2: Baseline characteristics of patients undergoing liver transplant.**

| **Author**  **Type of study** | **Transplant Reason** | **Groups (n)** | **MELD** | **Age** | **Operation time (mins)** | **Cold ischemia time (min)** | **Warm ischemia time (min)** | **Surgery** |
| --- | --- | --- | --- | --- | --- | --- | --- | --- |
| Beck-Shimmer et al.  RCT | End stage liver disease due to cirrhosis (55.10%)  Hepatocellular carcinoma (24.48%)  Others (21.42%) | Propofol (48) | 19 (11-24) | 53 (37-61) | 374 (319–444) | 414 (350-585) | 54 (40-65) | Deceased donor living transplantation. |
|  |  | Sevoflurane (50) | 17 (10-27) | 58 (51-64) | 365 (301–441) | 455 (352-553) | 54 (42-68) |  |
| Gajate et al. OBS | End stage liver disease due to cirrhosis (48.25%)  Hepatocellular carcinoma (43.78%)  Others (7.96%) | Propofol (143) | 17.4 [7.6] | 53.3  [8.4] | 410.9 [93.9] | 453.8  [122.5] | NR | Deceased donor living transplantation. |
|  |  | Sevoflurano (58) | 16.8  [7.1] | 54.0  [7.8] | 413.7 [105] | 471.2  [129.4] | NR |  |
| Lu et al.  OBS | End stage liver disease due to cirrhosis (94.59%)  Hepatocellular carcinoma (3.6%)  Other (1.8%) | Propofol (66) | 10.4 [3.0] | 52.4 [7.9] | 564 [126] | NR | NR | Deceased donor living transplantation.  Living donor liver transplantation. |
|  |  | Desflurane (45) | 10.0 [3.4] | 53.5 [8.3] | 540 [96] | NR | NR |  |
| Wu et al.  RCT | End stage liver disease due to cirrhosis (50%)  Hepatocellular carcinoma (50%) | Propofol (25) | 12.0 [5.5] | 52.0 [9.1] | 612.4 [89.3] | 70.1 [29.2] | 32.1 [18.0] | Living donor liver transplantation. |
|  |  | Desflurane (25) | 13.3 [6.8] | 53.2 [8.0] | 596.0 [95.5] | 74.0 [25.1] | 35.0 [16.3] |  |

Mean [SD]; Median (Range/IQR).

**1.3 Supplementary table 3: Liver donor hepatectomy supplementary table**

| **Author** | **Anesthesia  induction** | **Anesthesia maintenance** | | **Pharmacological conditioning* / Pringle maneuver**** |
| --- | --- | --- | --- | --- |
|  |  | **Propofol anesthesia group** | **Inhalational anesthesia group** |  |
| Ko et al. RCT | Propofol group:  Propofol TCI 6 mcg/ml.  Remifentanil TCI 4 ng/ml.  Vecuronium 0.15 mg/kg IV. | Propofol TCI 3-5 mcg/ml.  Remifentanil TCI 4 ng/ml.  BIS 40-50. | Desflurane:  - End-tidal Desflurane: 3.5-6.0%  - FiO2: 50%  BIS 40-50. | * The donor experienced pharmacological conditioning with desflurane through the entire procedure.  The same surgical team performed all operations, and either vascular clamping or the Pringle maneuver was not used |
|  | Inhalational anesthesia group:  Thiopental: 5 mg/kg IV.  Vecuronium 0.15 mg/kg IV. |  |  |  |
| Ozgul et al.  RCT | Propofol 2 mg/kg IV.  Remifentanil 1 mcg/kg IV.  Atracurium 0.6 mg/kg IV. | Propofol 10 mg/kg/h in the first 10 minutes, 8 mg/kg/h  in the following 10 minutes, and 6 mg/kg/h thereafter.  BIS 40-60.  Remifentanil 0.25 mcg/kg/min  Atracurium 0.5 mg/kg/h | Isoflurane:  - End-tidal Isoflurane: 0.5-1.5%  - FiO2 40%  - FGF 3 l/min  BIS 40-60.  Remifentanil 0.25 mcg/kg/min  Atracurium 0.5 mg/kg/h | * The donor experienced pharmacological conditioning with isoflurane through the entire procedure.  ** The same surgical team performed all operations using the Pringle maneuver routinely. |
| Rabie et al.  RCT | Propofol group  Propofol 2-3 mg/kg IV.  Sufentanil 0.2 mcg/kg IV.  Atracurium | Propofol 6-12 mg/kg/h.  Sufentanyl 0.2-0.4 mcg/kg/h.  Atracurium. | Isoflurane 0.8-1.2%.  FiO2 40-50%  Fentanyl 1-2 mcg/kg/h.  Atracurium. | * The donor experienced pharmacological conditioning with isoflurane through the entire procedure.  -*** |
|  | Isoflurane group  Propofol 2-3 mg/kg IV.  Fentanyl 2 mcg/kg IV.  Atracurium |  |  |  |
| Ucar et al.  RCT | Propofol 2 mg/kg IV.  Remifentanil 1 mcg/kg IV  Atracurium 0.6 mg/kg IV. | Propofol 10 mg/kg/h in the first 10 minutes, 8 mg/kg/h in the following 10 minutes, and 6 mg/kg/h thereafter.  BIS 40-60.  Remifentanil 0.25 mcg/kg/min.  Atracurium 0.5 mg/kg/h.  The propofol dose was increased when symptoms such as movement or grimacing were observed or when the BIS level was >60. | Isoflurane 0.5-1.5%.  FiO2 40%  BIS 40-60.  Remifentanil 0.25 mcg/kg/min.  Atracurium 0.5 mg/kg/h.  The isoflurane dose was increased when symptoms such as movement or grimacing were observed or when the BIS level was >60. | * The donor experienced pharmacological conditioning with isoflurane through the entire procedure.  ** “The objective of this study was to examine the effects of isoflurane and propofol on IR injury caused by the Pringle maneuver during donor hepatectomy” - The article does not specifically address the surgical technique used. |
|  | Thiopental: 5 mg/kg IV.  Remifentanil 1 mcg/kg IV  Atracurium 0.6 mg/kg IV. |  |  |  |

* Definition for pharmacological conditioning used: The cytoprotective effect of volatile anesthetics may either be initiated before the onset of ischemic injury (preconditioning), immediately on reperfusion (postconditioning), or for the entire surgical procedure (conditioning).

** The Pringle maneuver was included in our analyses as part of subgroup analyses to evaluate whether the use of inhalational anesthetics or propofol might exert a more pronounced effect in the context of ischemia-reperfusion. However, for studies that employed pharmacological preconditioning, we opted for stratified analyses, as the use of a specific anesthetic exclusively during the ischemic period could result in a modifying effect. Therefore, we did not jointly analyze studies that performed only the Pringle maneuver with those that combined the Pringle maneuver and pharmacological preconditioning. It is worth noting that in cases where pharmacological preconditioning was not specifically performed, continuous exposure to a single pharmacological agent throughout the surgical procedure after induction was considered pharmacological conditioning.

*** There is no description of whether the Pringle maneuver, any form of ischemic preconditioning, or inflow occlusion was performed.

**1.4 Supplementary table 4: Baseline characteristics of patients undergoing liver donor hepatectomy**

| **Author**  **Type of study** | **Groups (n)** | **ASA** | **Age** | **Operation time (mins)** | **Total liver volume** | **Graft volume** | **Remnant liver volume (%)** | **Surgery** |
| --- | --- | --- | --- | --- | --- | --- | --- | --- |
| Ko et al.  RCT | Propofol (35) | 1-2 | 30.6 [10.9] | 382.2 [47.3] | 1144.6 [237.8] | 696.9 [141.2] | 38.3 [9.8] | Living donor right hepatectomy |
|  | Desflurane (35) | 1-2 | 28.8 [8.6] | 376.3 [35.8] | 1171.7 [235.9] | 709.2 [174.1] | 38.7 [12.0] |  |
| Ozgul et al.  RCT | Propofol (40) | 1 | 31 (19–48) | 385 (240–540) | 1,150 (867–1,600) | 789 (497–1,190) | 34.1 (28.9–41.4) | Living donors right hepatectomy.  (18-65 years)  ASA 1 |
|  | Isoflurane (40) | 1 | 33 (18–61) | 360 (222–590) | 1,179.5 (879–1,520) | 759 (515–1,060) | 33.6 (29–42.2) |  |
| Rabie et al.  RCT | Propofol (10) | 1 | 24.6 [4.5] | 398.9 [70.3] | NR | NR | Remaining liver  volume was ≥32% in all donors. | Living donor right hepatectomy. |
|  | Isoflurane (10) | 1 | 26.8 [5.3] | 394.3 [60.8] | NR | NR |  |  |
| Ucar et al.  RCT | Propofol (29) | 1-2 | 30.7 [7.8] | 375.7 [71.2] | NR | 756.1 [171] | NR | Living donors hepatectomy.  (18-65 years)  ASA 1, 2 |
|  | Isoflurane (24) | 1-2 | 35.4 [10.5] | 363 [99.2] | NR | 692.5 [210.8] | NR |  |

Mean [SD]; Median (Range/IQR).

**1.5 Supplementary table 5: Liver mass resection supplementary table**

| **Author** | **Anesthesia  induction** | **Anesthesia maintenance** | | **Surgery** | **Pharmacological conditioning* / Pringle maneuver**** |
| --- | --- | --- | --- | --- | --- |
|  |  | **Propofol anesthesia group** | **Inhalational anesthesia group** |  |  |
| Beck-Shimmer et al. (2008)  RCT | Propofol TCI 4-6 mcg/ml.  Fentanyl: 3 mcg/kg IV.  Atracurium 0.5 mg/kg. | Propofol TCI 2-4 mcg/ml.  Fentanyl: 1-2 mcg/kg IV as needed.  Atracurium 5-10mg as needed  Remifentanil 0.3-0.6 mcg/kg/min IV. | Propofol TCI 2-4 mcg/ml.  Fentanyl: 1-2 mcg/kg IV as needed.  Atracurium 5-10mg as needed  Remifentanil 0.3-0.6 mcg/kg/min IV.    30 min before the induction of ischemia, propofol administration was stopped and replaced by sevoflurane (5-minute induction). Preconditioning involved 10 minutes of sevoflurane at 3.2 vol% (1.5 MAC). Sevoflurane was then discontinued, and propofol was reintroduced over 15 minutes (washout period). | Patients (>18 years) undergoing elective liver resection with inflow occlusion. | *Pharmacological preconditioning with sevoflurane was decided randomly and informed 30 minutes before portal triad clamping.  **Inflow occlusion was achieved by the tourniquet technique around the portal triad. Separate clamping of aberrant left hepatic arteries was carefully performed when present. |
| Kamel et al.  RCT | Propofol: 2 mg/kg IV.  Fentanyl: 2 mcg/kg IV.  Rocuronium: 0.6 mg/kg IV. | Propofol TCI 4 mcg/ml. BIS 40-60.  Fentanyl 1 mcg/kg IV boluses (as needed).  BIS > 60, then increase Ce by 0.5 mcg/ml.  Fentanyl: 2 mcg/kg/h 0-30 mins; 1.5 mcg/kg/h 31-150 mins; 1 mcg/kg/h 151 – 30 mins before closure. | Sevoflurane AGC:  - End-tidal Sevoflurane 2%;  - FiO2 40%;  - FGF 300 ml/min.  BIS 40-60.  Fentanyl 1 mcg/kg IV boluses (as needed).  BIS > 60, then increase Fi SEV by 0.2%.  Fentanyl: 2 mcg/kg/h 0-30 mins; 1.5 mcg/kg/h 31-150 mins; 1 mcg/kg/h 151 – 30 mins before closure. | Patients (18–60 years) with cirrhotic livers (ASA II, Child A) due to hepatitis C who were scheduled for elective liver resection. | * The patient experienced pharmacological conditioning with desflurane through the entire procedure.  -*** |
| Koraki et al.  RCT | Propofol: 2–2.5 mg/kg IV.  Fentanyl: 3 mcg/kg IV.  Lidocaine: 1 mg/kg IV.  Cisatracurium: 0.2 mg/kg IV. | Propofol: Continuous infusion at 0.05–0.1 mg/kg/min.  Fentanyl: 5 mcg/kg IV.  Cisatracurium: 2–4 mg IV bolus as needed.  Remifentanil: Continuous infusion at 0.3–0.6 mcg/kg/min. | Propofol: Continuous infusion at 0.05–0.1 mg/kg/min.  Fentanyl: 5 mcg/kg IV.  Cisatracurium: 2–4 mg IV bolus as needed.  Remifentanil: Continuous infusion at 0.3–0.6 mcg/kg/min.    30 min before the induction of ischemia, propofol administration was  stopped and replaced by desflurane.    Desflurane 1 MAC (induction time of 5 min). The pharmacological preconditioning was performed for 20 min, after which the following 5 min were used to cease desflurane administration and reinitiate propofol with washout of 5 min. | Patients older (≥18 years) undergoing  an elective extensive hepatic resection that included more than two segments of the liver. | *Pharmacological preconditioning with desflurane was decided randomly and informed 30 minutes before portal triad clamping.    ** Pringle Maneuver: Intermittent application with cycles of 10 minutes of inflow occlusion followed by 5 minutes of reperfusion, with a cumulative duration of at least 30 minutes. |
| Laviolle et al.  RCT | Propofol group:  Propofol TCI 4-8 mcg/ml.  Sufentanil 0.3-0.5 mcg/kg.  Atracurium 0.6 mg/kg. | Propofol TCI 3-6 mcg/ml.  During anesthesia, propofol and desflurane were titrated according to the clinical judgment of the anesthesiologist | Desflurane 0.7-1.0 MAC.  During anesthesia, propofol and desflurane were titrated according to the clinical judgment of the anesthesiologist | Adults older than 18 years hospitalized for partial hepatic resection of liver tumor (planned resection of four hepatic segments or less) requiring vascular hepatic occlusion were included. | * The patient experienced pharmacological conditioning with desflurane through the entire procedure.  **All patients underwent liver resection by the same trained surgeon under inflow vascular occlusion. It consisted in either intermittent complete pedicular clamping or selective hemi hepatic clamping. In case of complete pedicular clamping, the entire hepatic pedicle was encircled with a rubber tape to perform a Pringle maneuver with a tourniquet. Transection of the liver was performed under intermittent clamping by means of occlusion of blood inflow for 15 min and then release for 5 min.  Transection of the liver was performed under continuous clamping. |
|  | Inhalational anesthesia group:  Thiopental: 3-5 mg/kg.  Sufentanil 0.3-0.5 mcg/kg.  Atracurium 0.6 mg/kg. |  |  |  |  |
| Lisnyy et al.  OBS | Propofol 2 mg/kg IV.  Fentanyl 1-2 mcg/kg IV.  Atracurium 0.5 mg/kg IV. | Propofol infusion was continued at a dose sufficient for maintaining the BIS at the level of 40-60 | Propofol infusion was continued at a dose sufficient for maintaining the BIS at the level of 40-60  Preconditioning was provided 30 min prior to liver resection with sevoflurane inhalation (1.5—2 v/v% for 25—30 min) | Patients undergoing elective liver resection due to primary malignancy or liver metastasis | * The patient experienced pharmacological preconditioning with sevoflurane 30 minutes before liver resection  ** Intermittent pringle maneuver (20 min ischemia, 5 min reperfusion) was employed in 27 (65.9%) patients of Group Sevoflurane and 13 (40.6%) patients of Group Propofol |
| Matsumi et al.  RCT | Propofol, rocuronium, fentanyl, and remifentanil, was used for induction. | Propofol was infused by titrating for BIS values between 30 to 70 in the propofol group.  Epidural anesthesia. | Sevoflurane was administered by titrating the end-tidal concentration of sevoflurane between 0.6–2% for BIS values between 30 to 70 in the sevoflurane group.  Epidural anesthesia | Patients undergoing elective liver resection with the Pringle maneuver for metastatic or suspected metastatic hepatic masses. | * The patient experienced pharmacological conditioning with sevoflurane through the entire procedure.  **The Pringle maneuver was intermittently performed (cycles of 15 to 30 min of ischemia followed by 5 min of reperfusion). |
| Nguyen et al.  OBS | Propofol 2-3 mg/kg.  Sufentanil 0.5 mcg/kg.  Atracurium 0.5 mg/kg. | Propofol TCI | Sevoflurane  - End-tidal Sevoflurane 1.2-2.5%;  - FiO2 40%; | Patients older than 18 years undergoing any kind of liver resection with intermittent portal triad occlusion for benign or malignant diseases | * The patient experienced pharmacological conditioning with sevoflurane through the entire procedure.  **After liver mobilization, intermittent clamping was realized by the tourniquet around the portal triad (Pringle maneuver). Intermittent clamping was repeated until total completion of hepatic transection. |
|  |  |  | 30 min before starting intermittent clamping, 1.5 MAC of sevoflurane was applied for 15 min followed by a 15 min washout. |  | * The patient experienced pharmacological preconditioning with sevoflurane 30 minutes before portal triad clamping.  **After liver mobilization, intermittent clamping was realized by the tourniquet around the portal triad (Pringle maneuver). Intermittent clamping was repeated until total completion of hepatic transection. |
| Rodríguez et al.  RCT | The same standardized  anesthetic protocol was used to manage all patients  included in the study  Propofol 2-3 mg/kg  Fentanyl 2 mcg/kg  Atracurium 0.5 mg/kg | Propofol TCI  BIS 40-60  Remifentanil 0.1-0.3 mcg/kg  Fentanyl boluses  Atracurium 0.3 mcg/kg/min | Propofol TCI  BIS 40-60  Remifentanil 0.1-0.3 mcg/kg  Fentanyl boluses  Atracurium 0.3 mcg/kg/min  20 min before starting intermittent clamping, 1.5 MAC of sevoflurane was applied for 15 min followed by a 15 min washout. | Patients undergoing elective partial liver resection under intermittent Pringle  maneuver | * The patient experienced pharmacological preconditioning with sevoflurane 20 minutes before portal triad clamping.  ** Intermittent Pringle maneuvers of 15-minute occlusion of the hepatic pedicle and 5-minute clamp-free intervals were performed |
| Slankamenac et al.  OBS | “Both groups received the same induction of anaesthesia according to the standardized procedures with fentanyl and atracurium as boluses according to clinical need and remifentanil.” | Propofol TCI 2-4 mcg/ml. | Sevoflurane 1.0–2.5%. | Patients undergoing any type of liver resection with inflow occlusion for  benign or malignant diseases. | * The patient experienced pharmacological conditioning with sevoflurane through the entire procedure.  ** Inflow occlusion: The parenchymal transections were done with the Kelly clamp crushing technique under an inflow occlusion procedure. The tourniquet technique around the portal triad was used as inflow occlusion (Pringle maneuver). |
| Song et al.  RCT | Propofol TCI 4-6 mcg/ml  Fentanyl 3 mcg/kg  Cisatracurium 0.2 mg/kg | Propofol TCI 4-6 mcg/ml.  BIS 35-45  Fentanyl 1 to 2 kg/kg as needed.  Cisatracurium 5 to 10 mg as needed. | Sevoflurane 1.5-2.5%  BIS 35-45  Fentanyl 1 to 2 kg/kg as needed.  Cisatracurium 5 to 10 mg as needed. | Patients undergoing hepatectomy with inflow occlusion.  ≥ 18 years  ASA 1, 2, 3 | * The patient experienced a pharmacological conditioning with sevoflurane through the entire procedure  ** After mobilization of the liver, inflow occlusion was achieved by the tourniquet technique around the portal triad (Pringle maneuver). The length of time for continuous inflow occlusion was determined by the surgeons. |
|  | Sevoflurane 8%  Fentanyl 3 mcg/kg  Cisatracurium 0.2 mg/kg |  |  |  |  |
| Yang et al.  RCT | Thiopental: 3-5 mg/kg IV.  Remifentanil 1.5 mcg/kg IV  Cisatracurium 0.15 mg/kg IV. | Propofol TCI 3-6 mcg/ml.  Cisatracurium 10 mg as needed.  Epidural anesthesia. | Isoflurane 1–2%  Cisatracurium 10 mg as needed.  Epidural anesthesia. | Patients (18–80 years) with hepatitis B related cirrhosis (ASA 2-3, Child A) who were scheduled for elective liver resection. | * The patient experienced a pharmacological conditioning with isoflurane through the entire procedure  ** Liver resection was carried out with intermittent clamping of the portal triad for 15 min and then releasing it for 5 min. |
| Yassen et al.  RCT | Propofol: 2 mg/kg IV.  Fentanyl: 1 mcg/kg IV.  Rocuronium: 1 mg/kg IV. | Propofol TCI 3-4 mcg/ml  Fentanyl 2 mcg/kg/h for 30 min, 1.5 mcg/kg/h from 31-150 min, and 1 mcg/kg/h until 30 min before skin closure | Desflurane (“1 l/min mixture of air, oxygen  and Desflurane (ETCO2 32-36 mmHg)”)  BIS 40-60 | Adult patients (≥21 years) with cirrhosis (Child A) admitted for major liver resection. | * The patient experienced a pharmacological conditioning with desflurane through the entire procedure  “Selective vascular occlusion of hepatic inflow was not adopted by the surgeons in our study” |
|  | Propofol TCI 3-4 mcg/ml  Fentanyl 3 mcg/kg  Rocuronium: 1 mg/kg IV. |  |  |  |  |

* Definition for pharmacological conditioning used: The cytoprotective effect of volatile anesthetics may either be initiated before the onset of ischemic injury (preconditioning), immediately on reperfusion (postconditioning), or for the entire surgical procedure (conditioning).

** The Pringle maneuver was included in our analyses as part of subgroup analyses to evaluate whether the use of inhalational anesthetics or propofol might exert a more pronounced effect in the context of ischemia-reperfusion. However, for studies that employed pharmacological preconditioning, we opted for stratified analyses, as the use of a specific anesthetic exclusively during the ischemic period could result in a modifying effect. Therefore, we did not jointly analyze studies that performed only the Pringle maneuver with those that combined the Pringle maneuver and pharmacological preconditioning. It is worth noting that in cases where pharmacological preconditioning was not specifically performed, continuous exposure to a single pharmacological agent throughout the surgical procedure after induction was considered pharmacological conditioning.

*** There is no description of whether the Pringle maneuver, any form of ischemic preconditioning, or inflow occlusion was performed.

**1.6 Supplementary table 6: Baseline characteristics of patients undergoing liver mass resection**

| **Author**  **Type of study** | **Groups (n)** | **ASA** | **Age** | **Operation time – mins** | **Ischemia time – mins** | **Cirrhosis** | **Baseline AST – U/l** | **Baseline ALT – U/l** | **Baseline Bilirubin – mg/dl** | **Indication for surgery (%)** | **Extent of Hepatectomy (%)** |
| --- | --- | --- | --- | --- | --- | --- | --- | --- | --- | --- | --- |
| Beck-shimmer et al. (2008)  RCT | Propofol (34) | 1/2/3 | 57.82 [12.82] | 267.65 [95.72] | 35.12 [6.32] | 0% | 34.21 [20.86] | 39.91 [43.25] | 0.69 [0.65] | Hepatic cancer: 4.68%  Liver metastasis: 51.56%  Others: 43.75% | Major resection 43.75% (≥ 3 segments) |
|  | Sevoflurane (30) | 1/2/3 | 54.23 12.74] | 259.83 [87.81] | 36.03 [5.62] | 0% | 32.83 [15.67] | 32.53 [20.15] | 0.75 [0.62] |  |  |
| Kamel et al.  RCT | Propofol (25) | 2 | 55 (51-59) | 205 (185–240) | NR | 100% | NR | NR | NR | Patients with hepatitis C related cirrhosis scheduled for elective liver resection. | Caudate lobe hepatectomy 4%  Right formal hepatectomy 14%  Left formal hepatectomy 8%  Left lateral hepatectomy 24%  Non anatomical hepatectomy 50% |
|  | Sevoflurane (25) | 2 | 56 (50-60) | 180 (175–206) | NR | 100% | NR | NR | NR |  |  |
| Koraki et al.  RCT | Propofol (23) | NR | 61.5 [11,4] | 270 (210-340) | 55 [26] | 0% | NR | NR | NR | Hepatic cancer: 41.30%  Liver metastasis: 43.47%  Others: 15.21% | Right extended 39.13%  Right 39.13%  Left 39.13%  Left extended 10.86% |
|  | Propofol and Desflurane (23) | NR | 64.5 [10.6] | 300 (241-380) | 61 [30] | 0% | NR | NR | NR |  |  |
| Laviolle et al.  RCT | Propofol (17) | NR | 60 [14] | NR | 39 [23] | 0% | 39 [25] | 33 [21] | NR | Partial hepatic resection of liver tumor (planned resection of four hepatic segments or less) | Number of hepatic segments resected:  1: 2 (6.66%)  2: 6 (20%)  3: 5 (16.66%)  4: 16 (53.33%)  5: 1 (3.33%) |
|  | Desflurane (13) | NR | 61 [13] | NR | 40 [32] | 0% | 32 [12] | 34 [25] | NR |  |  |
| Lisnyy et al.  OBS | Propofol (32) | 2-3 | 58 [7.4] | NR | 31 [15] | NR | 36.6 [16.9] | 34.2 [21] | 0.84 [0.65] | Hepatic cancer: 4.1%  Liver metastasis: 84.93%  Others: 10.95% | Major hepatectomy 21.91% (≥3 anatomical segments) |
|  | Sevoflurane (41) | 2-3 | 58 [6.4] | NR | 45 [17 ] | NR | 33.6 [15.9] | 31.6 [20] | 0.75 [0.40] |  |  |
| Matsumi et al.  RCT | Propofol (28) | 2/3 | 66.3 [12.3] | 237.4 [46.6] | 59.7 [27.0] | 0% | 26.4 [12.3] | 24.0 [13.7] | 0.8 [0.2] | Patients diagnosed with metastatic or suspected metastatic hepatic masses undergoing elective liver resection | Does not specify the extent of the hepatectomy performed. |
|  | Sevoflurane (28) | 2/3 | 64.7 [10.1] | 290.4 [98.2] | 68.5 [42.7] | 0% | 27.4 [12.3] | 24.9 [19.9] | 0.8 [0.4] |  |  |
| Nguyen et al.  OBS | Propofol (26) | NR | 63 (53–68) | NR | 33 (23–50) | 11.53% | 29 (22–34) | 29 (19–37) | 0.47 (0.29–0.64) | Hepatic cancer: 20%  Liver metastasis: 50%  Others: 30% | Major hepatectomy 38.33% (>3 segments) |
|  | Propofol and Sevoflurane (27) | NR | 65 (57–68) | NR | 37 (16–44) | 7.4% | 28 (20–60) | 30 (20–49) | 0.47 (0.29–0.76) |  |  |
|  | Sevoflurane (67) | NR | 61 (51–67) | NR | 40 (27–61) | 14.92% | 26 (19–38) | 22 (16–37) | 0.47 (0.29–0.59) |  |  |
| Rodríguez et al.  RCT | Propofol (36) | 1-3 | 62 [20] | 300 [60] | 52.6 [29.3] | 0% | 24 [16] | 21 [16] | 0.5 [0.21] | Hepatic cancer: 8%  Liver metastasis: 80%  Others: 12% | Major hepatectomy 52.85% (>3 segments) |
|  | Sevoflurane (34) | 2-3 | 65 [12] | 300 [64] | 46.3 [26.6] | 0% | 22 [15] | 20 [15] | 0.5 [0.33] |  |  |
| Slankamenac et al.  OBS | Propofol (86) | 1/2/3/4 | 56.3 [12.7] | 279.6 [121.6] | 33.4 [8.5] | 0% | 45.7 [49.4] | 51.9 [67.1] | 0.95 [1.8] | Hepatic cancer: 16.74%  Liver metastasis: 32.15%  Others: 51.10% | Major resection 47.13% |
|  | Sevoflurane (141) | 1/2/3/4 | 59.2 [14.8] | 289.1 [133.4] | 35.5 [13.6] | 0% | 50.1 [47.8] | 63.4 [93.7] | 1.1 [2.2] |  |  |
| Song et al.  RCT | Propofol (50) | 1/2/3 | 51.4 [7.8] | 124.3 [29.1] | 18.4 [6.3] | 54% | 46 [29] | 43 [26] | 0.79 [0.35] | Hepatic cancer: 72%  Others: 28% | Major resection 45% |
|  | Sevoflurane (50) | 1/2/3 | 48.5 [8.9] | 136.0 [38.4] | 21.4 [8.5] | 66% | 36 [11] | 40 [13] | 0.85 [0.30] |  |  |
| Yang et al.  RCT | Propofol (30) | 2/3 | 53.6 [9.5] | NR | 15 [2] | 100% | 44 [23] | 34 [32] | 0.82 [0.12] | Patients with hepatitis B related cirrhosis scheduled for resection of hepatocellular carcinoma | Does not specify the extent of the hepatectomy performed. |
|  | Isoflurane (30) | 2/3 | 52 [9.1] | NR | 15 [5] | 100% | 34 [14] | 38 [21] | 0.70 [0.18] |  |  |
| Yassen et al.  RCT | Propofol (25) | NR | 55.2 [12.1] | NR | - | 100% | 37.68 [6.07] | 43.88 [9.82] | - | Adult patients with cirrhosis admitted for major liver resection. | Major elective liver resection |
|  | Desflurane (25) | NR | 53.6 [10.4] | NR | - | 100% | 36.32 [2.64] | 43.36 [5.50] | - |  |  |

Mean [SD]; Median (Range/IQR).

2.1 Propofol versus inhalational anesthesia in liver transplant patients

2.1.1 Supplementary figure 1: ALT 1st postoperative day


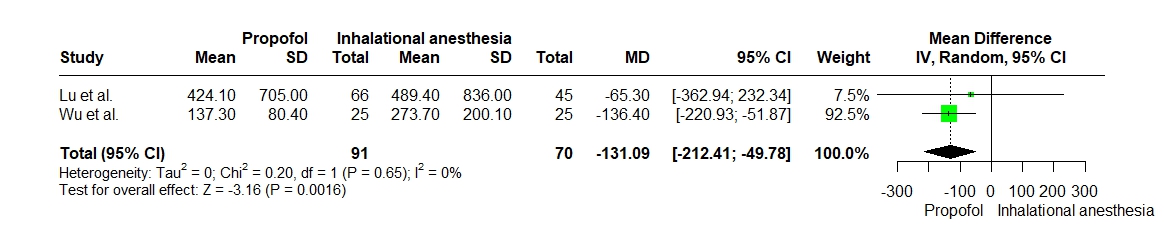


2.1.2 Supplementary figure 2: Length of hospital stay (days)


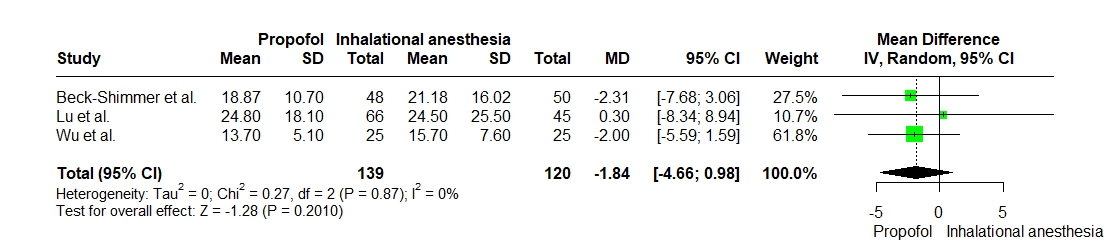


2.1.3 Supplementary figure 3: Length of hospital stay (days) - Leave-one-out
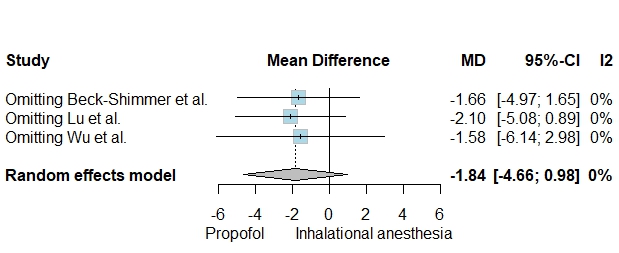


2.1.4 Supplementary figure 4: Length of ICU stay (hours)


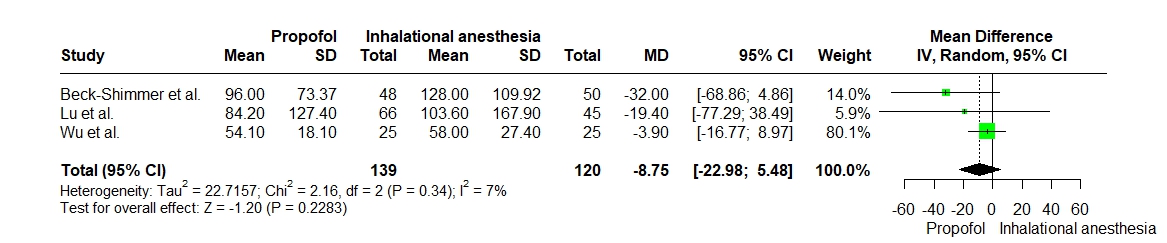


2.1.5 Supplementary figure 5: Length of ICU stay (hours) - Leave-one-out
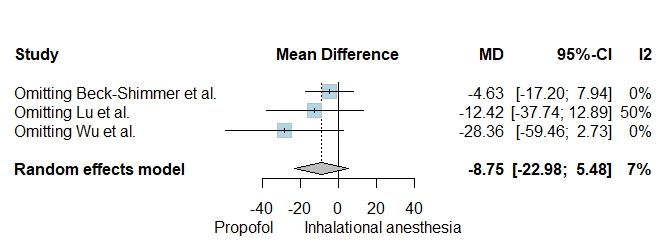


2.1.6 Supplementary figure 6: Packed red blood cell units


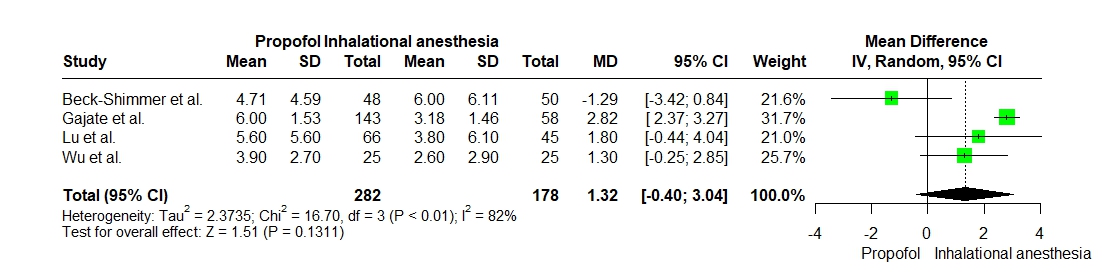


2.1.7 Supplementary figure 7: Packed red blood cell units - Leave-one-out


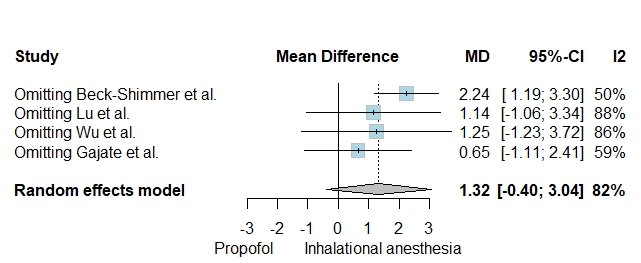


2.1.8 Supplementary figure 8: Fresh Frozen Plasma units


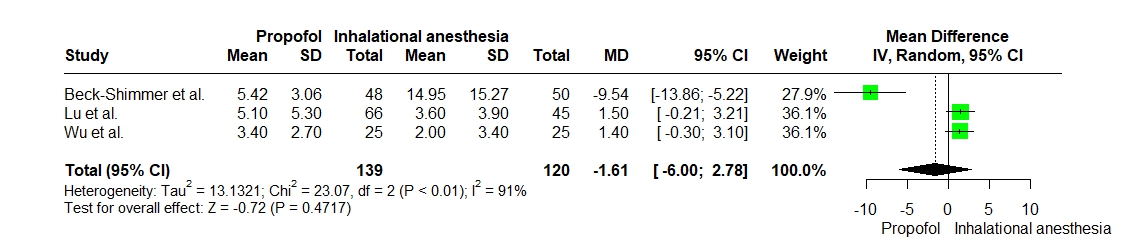


2.1.9 Supplementary figure 9: Fresh Frozen Plasma units - Leave-one-out
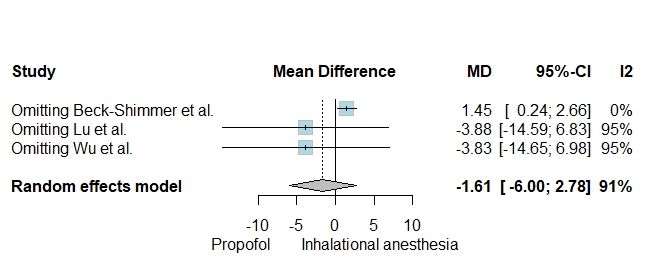


2.1.10 Supplementary figure 10: Platelets units
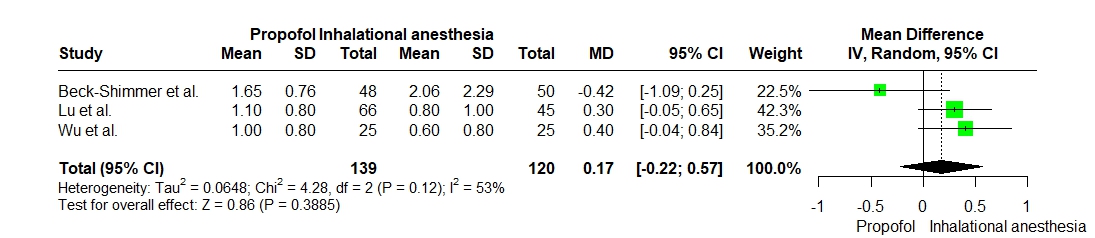


2.1.11 Supplementary figure 11: Platelets units - Leave-one-out
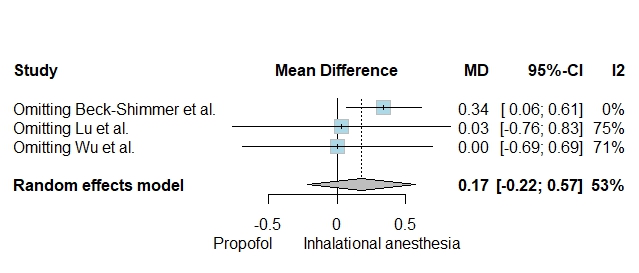


2.1.12 Supplementary figure 12: Total fluid infusion (L)
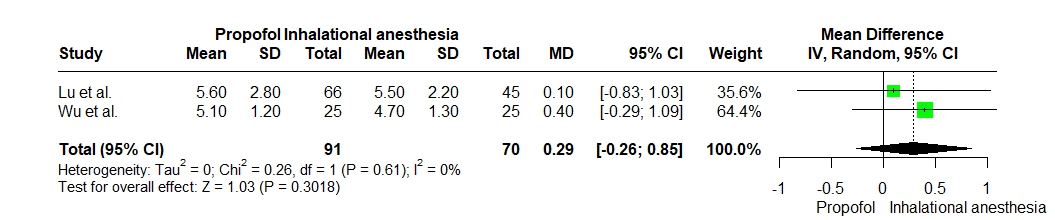


2.1.13 Supplementary figure 13: Estimated blood loss (L)
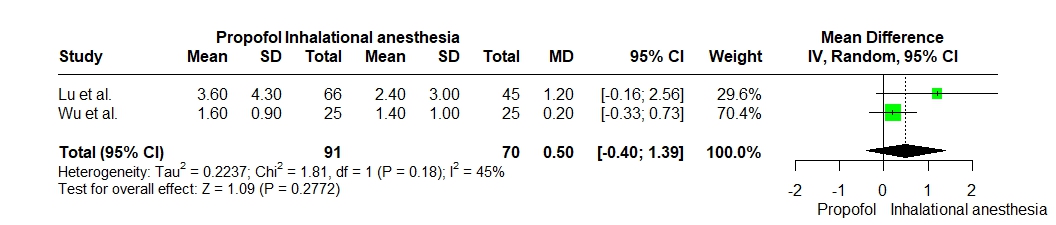


2.1.14 Supplementary figure 14: Urine output (L)


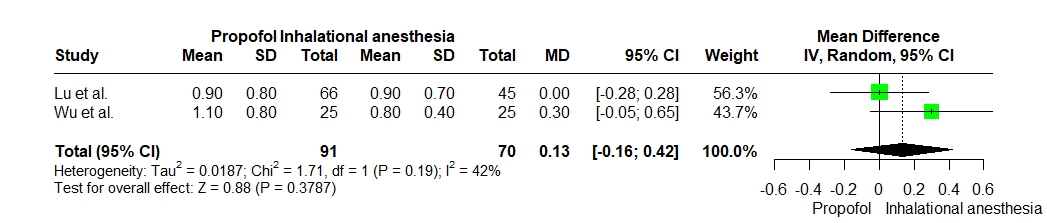


2.1.15 Supplementary figure 15: Early allograft dysfunction


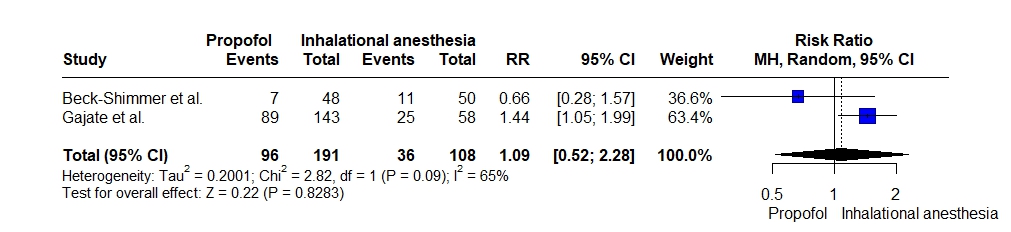


2.2 Propofol versus inhalational anesthesia in liver donor hepatectomy

2.2.1 Supplementary figure 16: Total fluid infusion (ml)
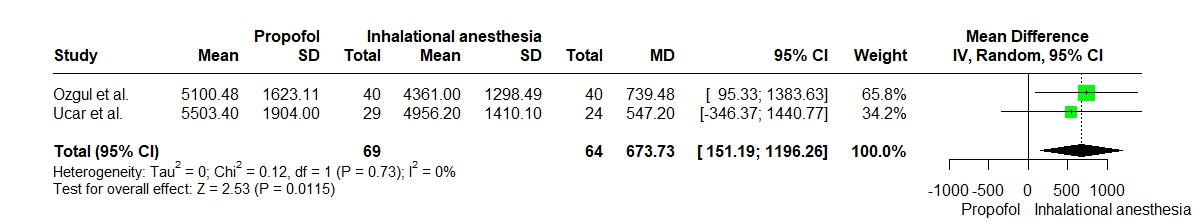


2.2.2 Supplementary figure 17: Estimated blood loss (ml)
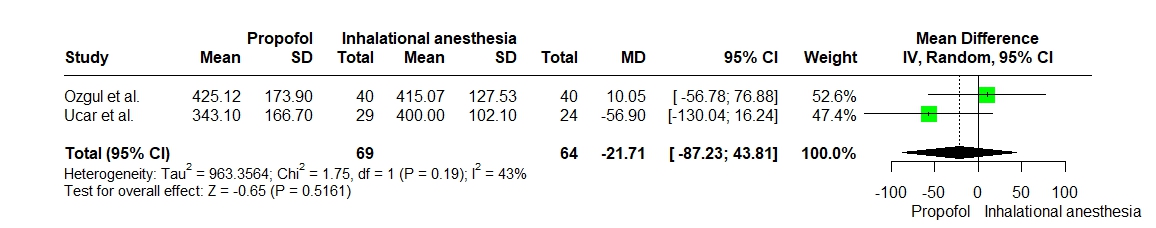


2.2.3 Supplementary figure 18: Urine output (ml)
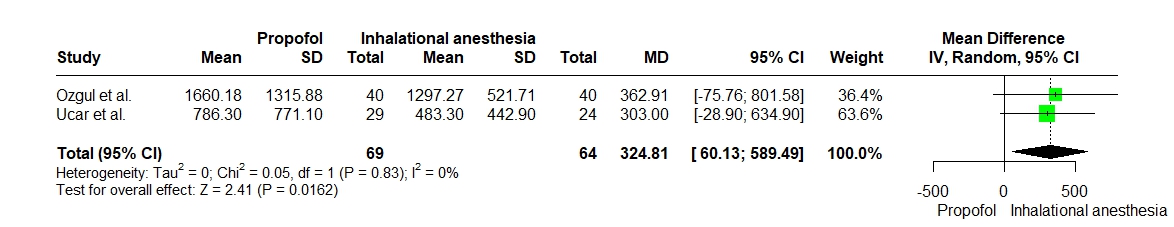


2.3 Propofol versus inhalational anesthesia in liver mass resection with pharmacological preconditioning

2.3.1 Supplementary figure 19: Peak AST
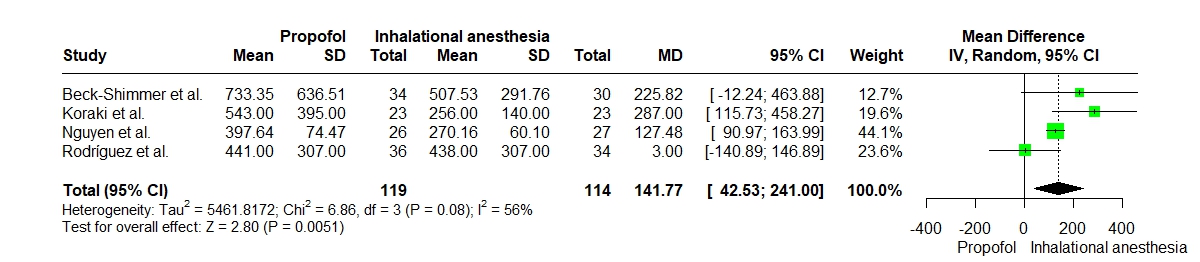


2.3.2 Supplementary figure 20: Peak AST - Leave-one-out
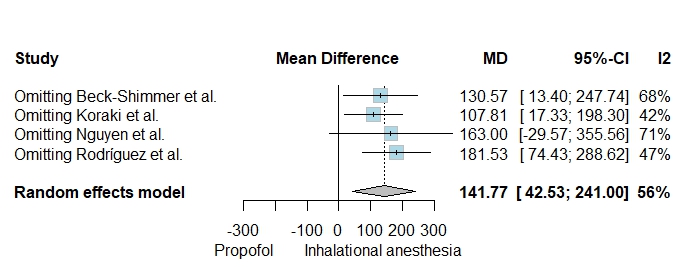


2.3.3 Supplementary figure 21: Peak ALT
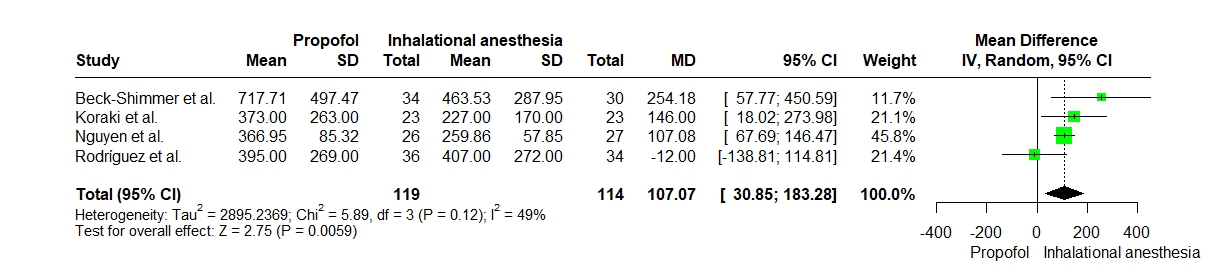


2.3.4 Supplementary figure 22: Peak ALT - Leave-one-out
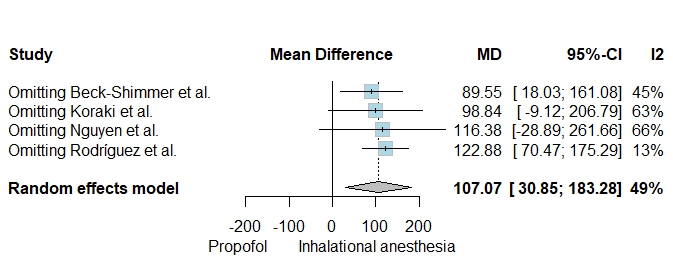


2.3.5 Supplementary figure 23: Peak Bilirubin
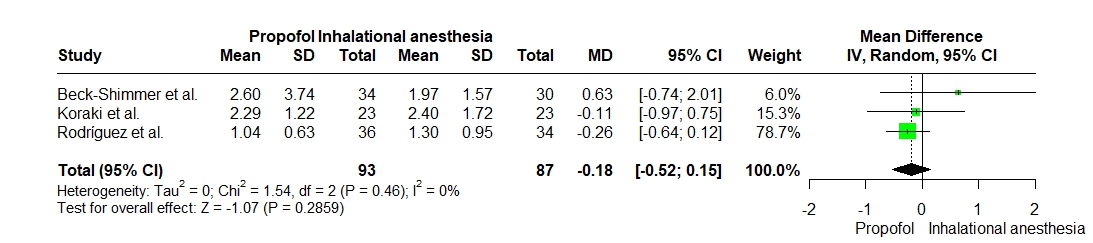


2.3.6 Supplementary figure 24: Peak Bilirubin - Leave-one-out
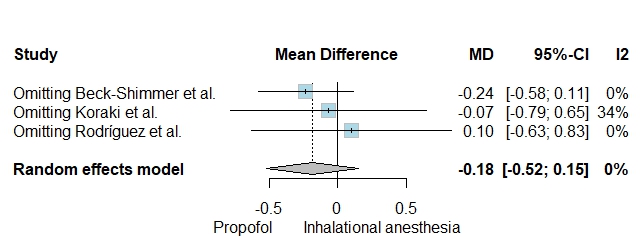


2.3.7 Supplementary figure 25: Length of hospital stay (days)


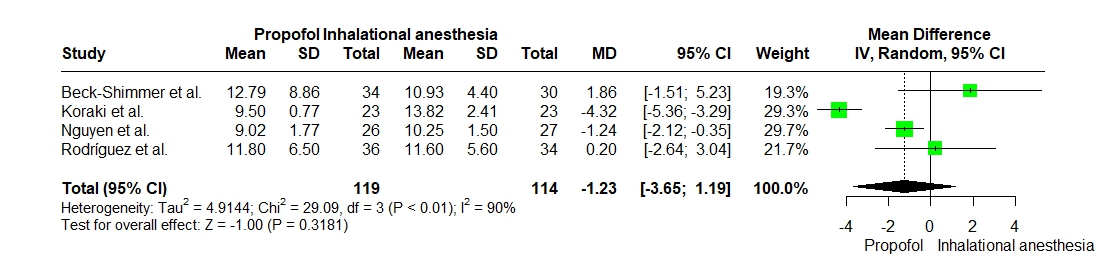


2.3.8 Supplementary figure 26: Length of hospital stay (days) - Leave-one-out
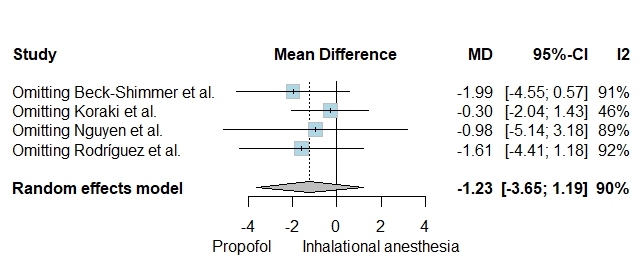


2.3.9 Supplementary figure 27: Estimated blood loss (ml)
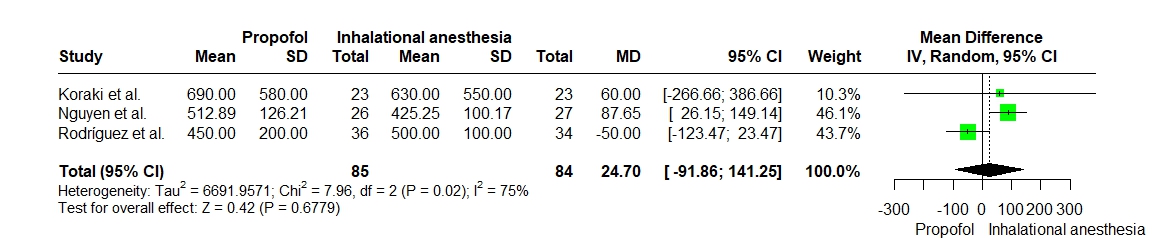


2.3.10 Supplementary figure 28: Estimated blood loss (ml) - Leave-one-out
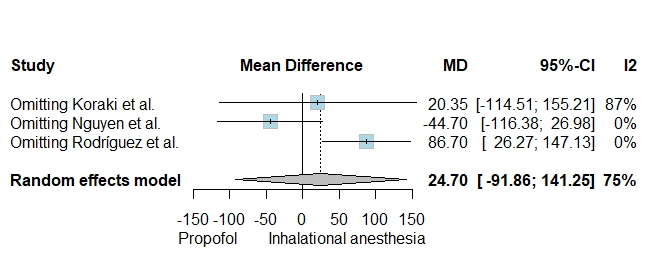


2.4 Propofol versus inhalational anesthesia in liver mass resection

2.4.1 Supplementary figure 29: Peak AST
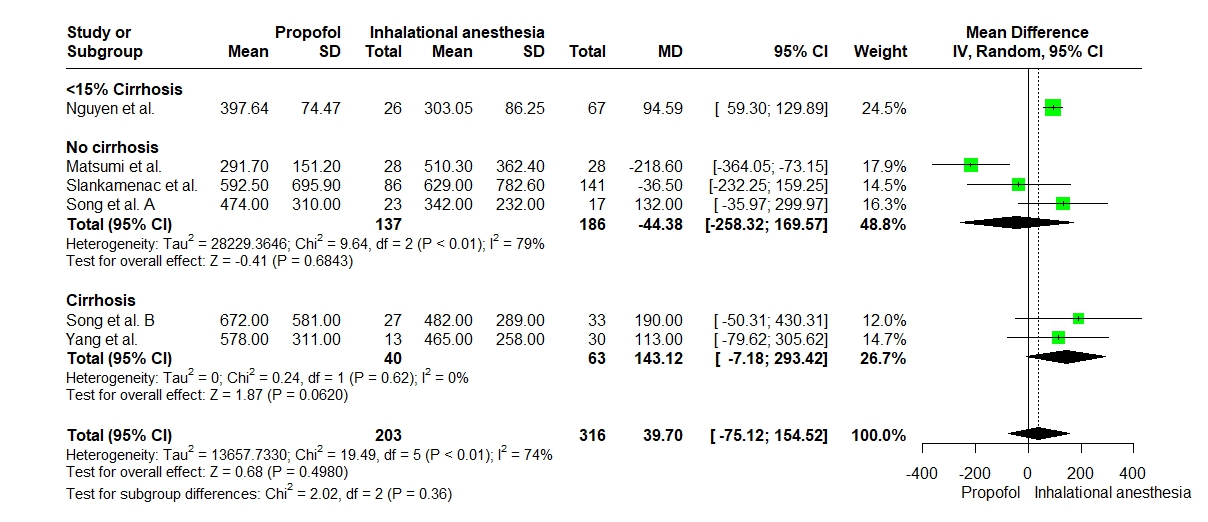


2.4.2 Supplementary figure 30: Peak AST - Leave-one-out
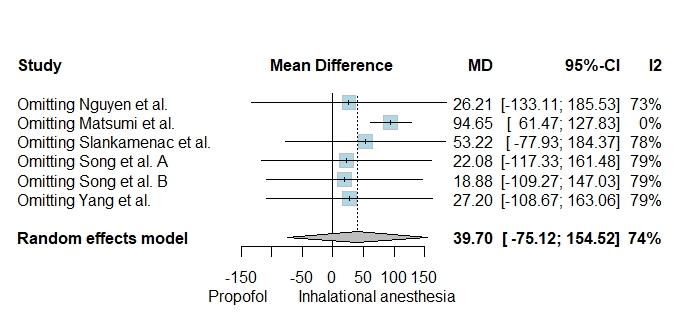


2.4.3 Supplementary figure 31: Peak ALT
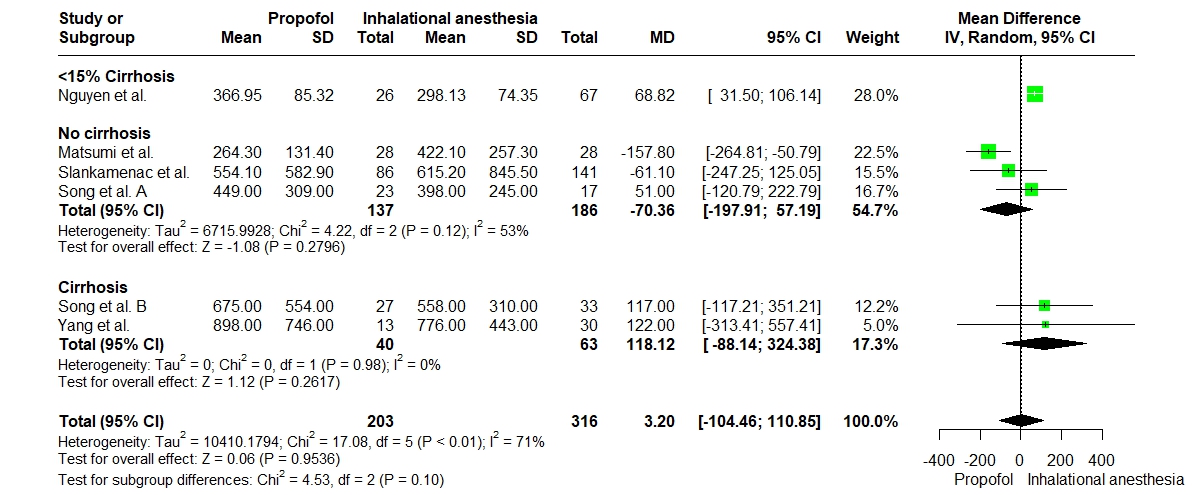


2.4.4 Supplementary figure 32: Peak ALT - Leave-one-out
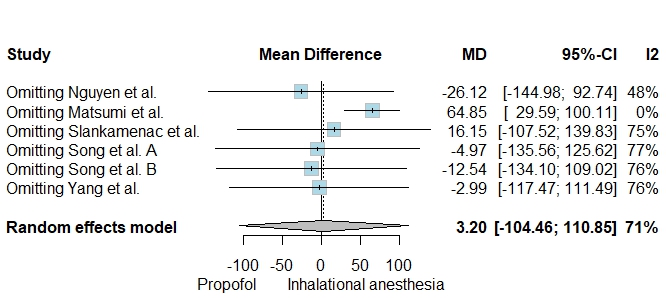


2.4.5 Supplementary figure 33: Peak Bilirubin
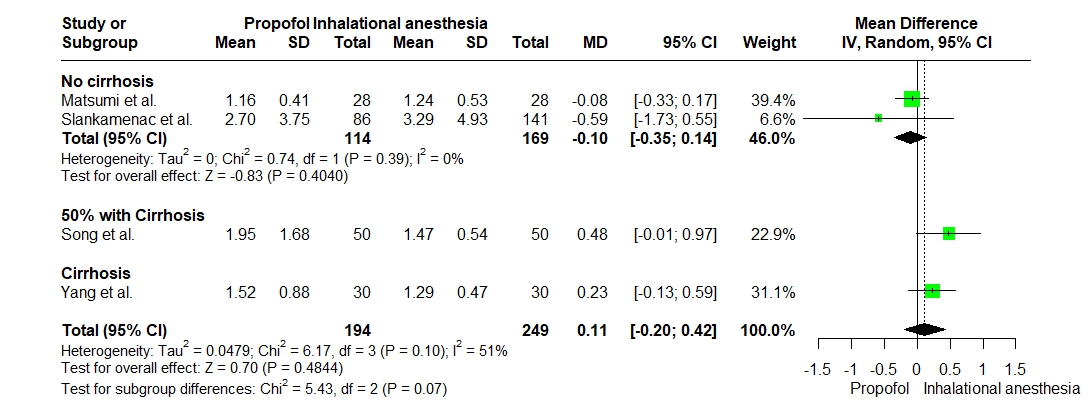


2.4.6 Supplementary figure 34: Peak Bilirubin - Leave-one-out
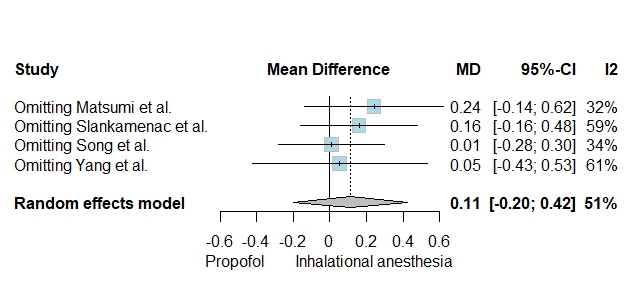


2.4.7 Supplementary figure 35: AST 1st postoperative day
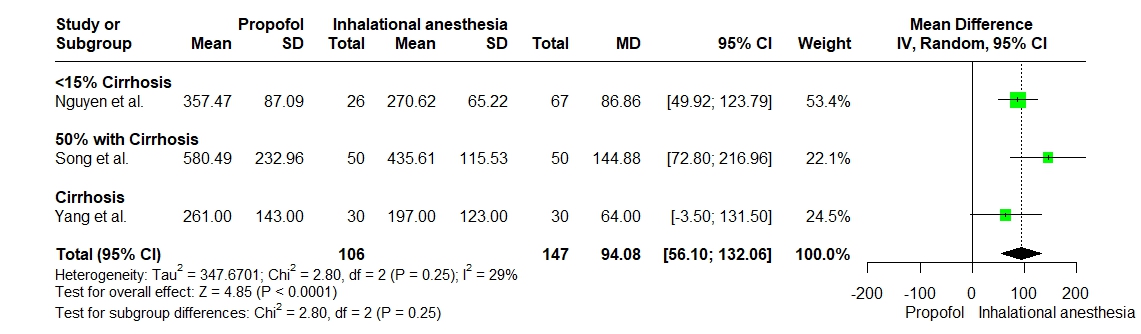


2.4.8 Supplementary figure 36: AST 1st postoperative day - Leave-one-out
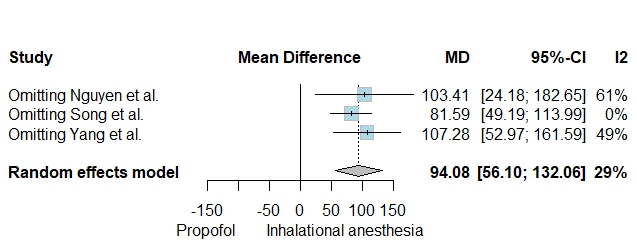


2.4.9 Supplementary figure 37: AST 3rd postoperative day
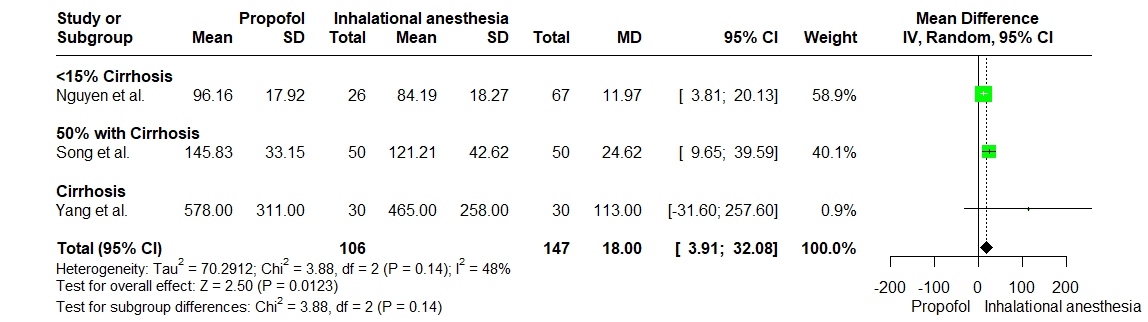


2.4.10 Supplementary figure 38: AST 3rd postoperative day - Leave-one-out
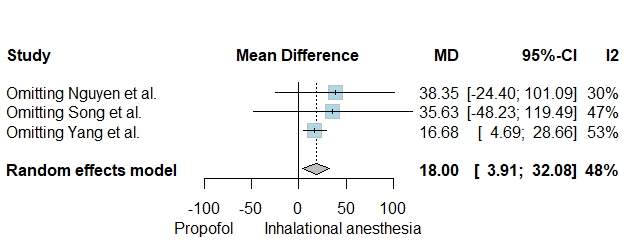


2.4.11 Supplementary figure 39: ALT 1st postoperative day
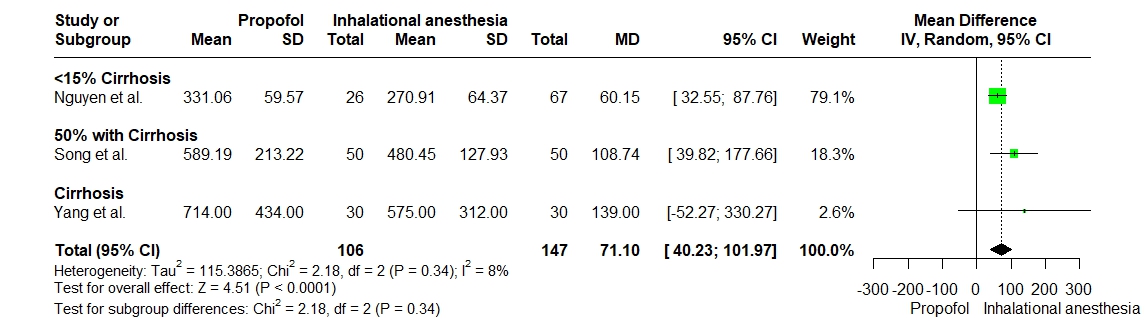


2.4.12 Supplementary figure 40: ALT 1st postoperative day - Leave-one-out
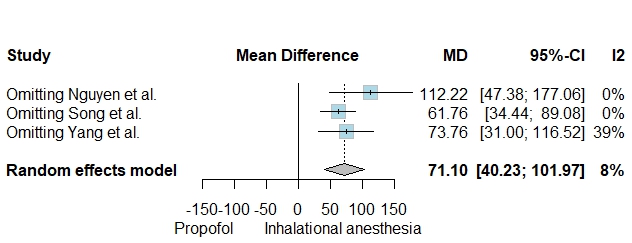


2.4.13 Supplementary figure 41: ALT 3rd postoperative day
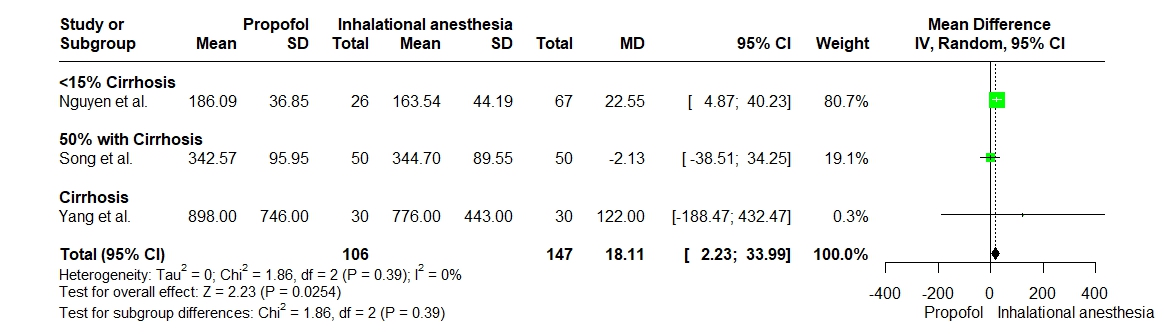


2.4.14 Supplementary figure 42: ALT 3rd postoperative day - Leave-one-out
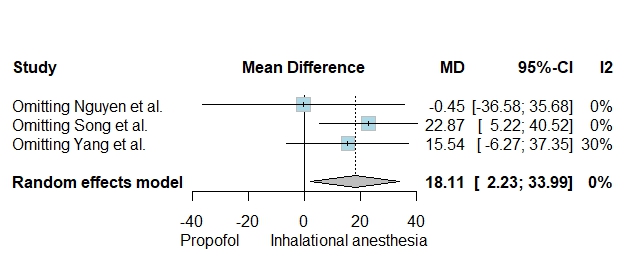


2.4.15 Supplementary figure 43: Length of hospital stay (days)
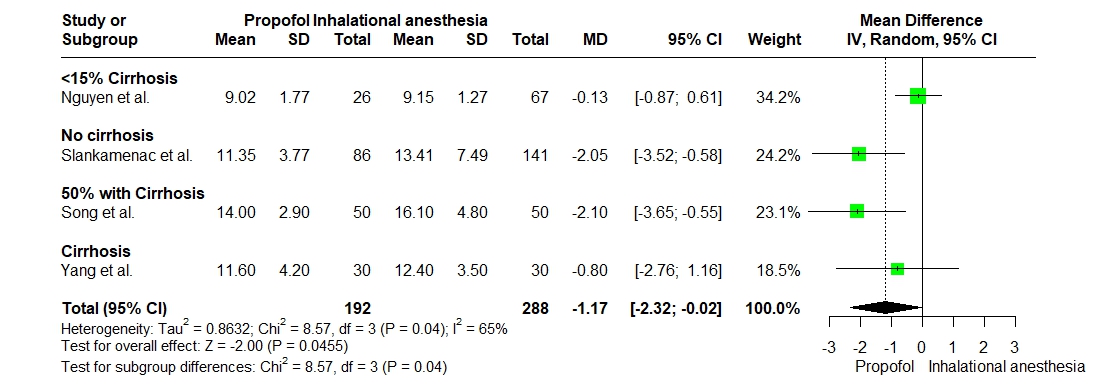


2.4.16 Supplementary figure 44: Length of hospital stay (days) - Leave-one-out
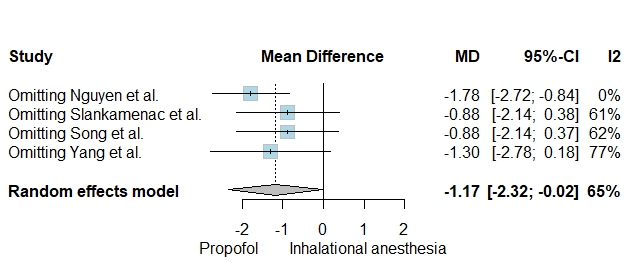


2.4.17 Supplementary figure 45: Total fluid infusion (ml)
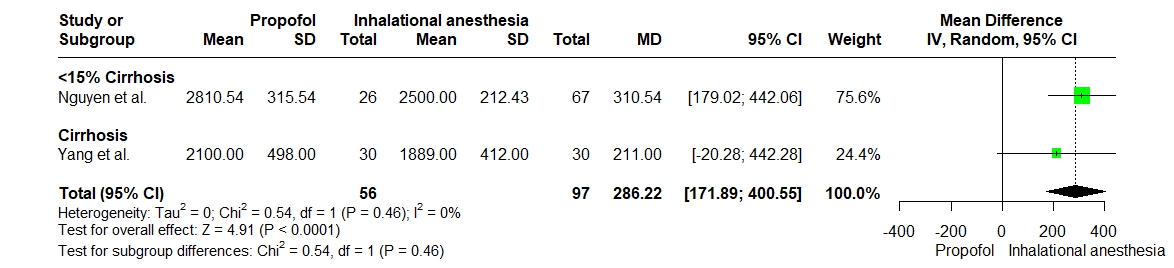


2.4.18 Supplementary figure 46: Estimated blood loss (ml)
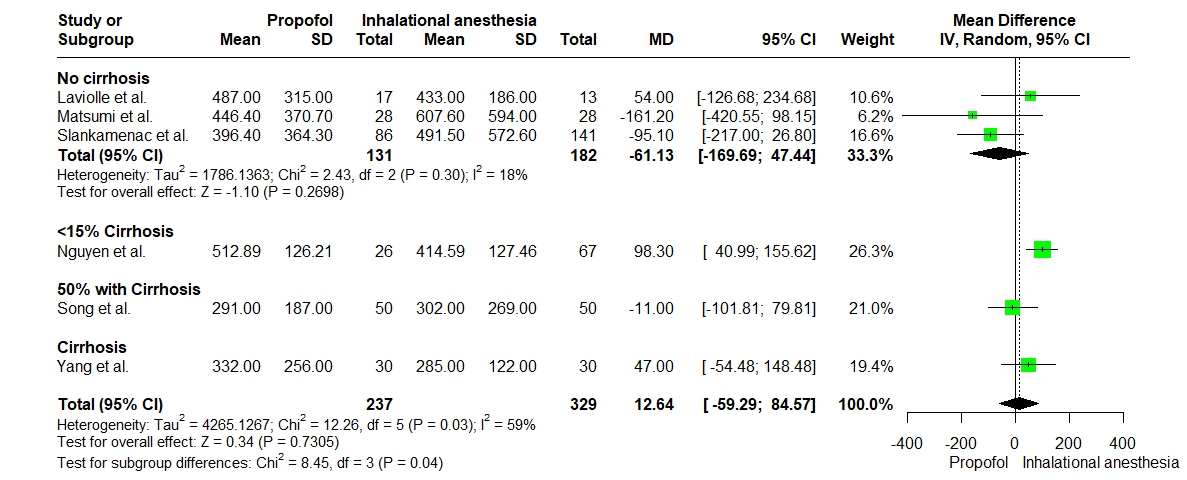


2.4.19 Supplementary figure 47: Estimated blood loss (ml) - Leave-one-out
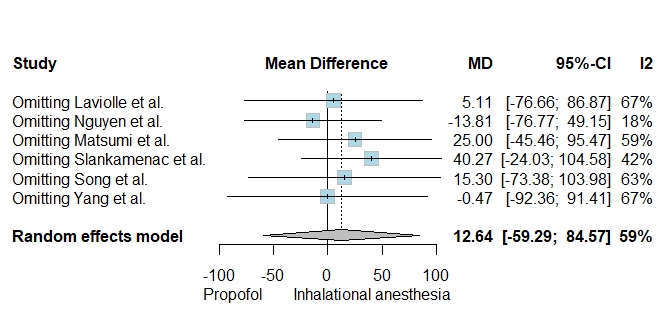


2.5 Risk of bias assessment

2.5.1 Supplementary figure 48: Risk of bias assessment


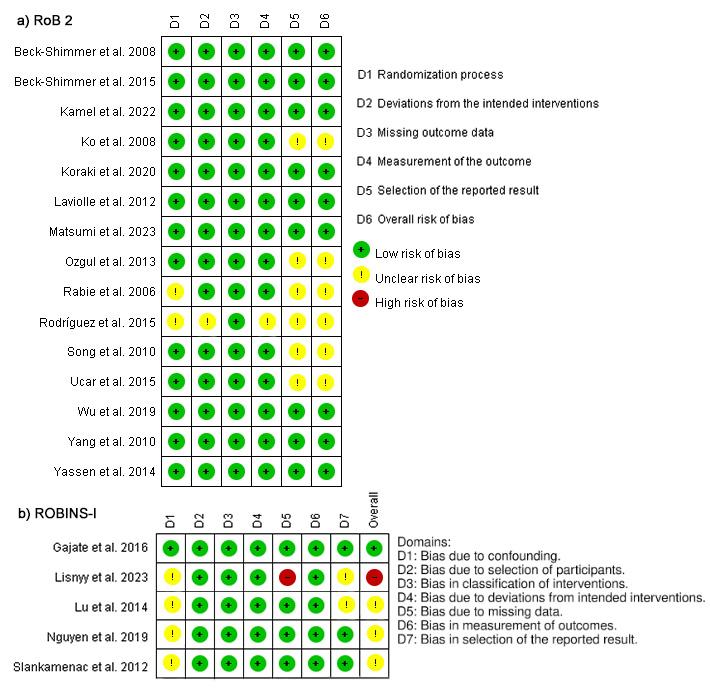

Supplement: Supplementary file 1 [file mmc1.docx]
